# Effect of transcranial direct current stimulation over the primary motor cortex on short-term balance acquisition in healthy individuals

**DOI:** 10.1186/s12984-025-01663-3

**Published:** 2025-07-04

**Authors:** Ömer Burak Tor, Michael A. Nitsche, Edmund Wascher, Nevin A. Guzel, Charles S. Layne

**Affiliations:** 1 Department of Physiotherapy and Rehabilitation, Faculty of Health Sciences, Balıkesir University, Çağış Campus, Balıkesir, Türkiye; 2 Department of Psychology and Neurosciences, Leibniz Research Centre for Working Environment and Human Factors, Ardeystraße 67, 44139 Dortmund, Germany; 3 Department of Physiotherapy and Rehabilitation, Gazi University, Ankara, Türkiye; 4 University Clinic of Psychiatry and Psychotherapy, Protestant Hospital of Bethel Foundation, Bielefeld, Germany; 5 Department of Ergonomics, Leibniz Research Centre for Working Environment and Human Factors, Dortmund, Germany; 6 German Center for Mental Health (DZPG), Bochum, Germany; 7 Department of Health and Human Performance, University of Houston, Houston, TX USA; 8 Center for Neuromotor and Biomechanics Research, Houston, TX USA

**Keywords:** Primary motor cortex, Transcranial direct current stimulation, Balance training, Balance learning, Postural perturbations, Electroencephalography, Transcranial magnetic stimulation

## Abstract

**Background:**

The primary motor cortex (M1) is central to motor learning processes, and an increasing number of studies have suggested its role in balance control. However, the specific role of M1 in balance control remains unclear, and a causal contribution to improvements in balance ability after balance training has not yet been proven. Transcranial direct current stimulation (tDCS) is a non-invasive brain stimulation technique that modifies brain activity and enables to probe the involvement of M1 in balance learning. The current study aims to explore the role of M1 in the acquisition of balance skills by applying tDCS during short-term perturbation-based balance training.

**Methods:**

Thirty-four participants were randomly assigned to one of three groups receiving balance training combined with tDCS: anodal tDCS, sham tDCS, and a control group without stimulation. All participants were involved in a structured, three-session perturbation-based balance training program completed within one week. During these sessions, the assigned tDCS protocol was applied over the M1 leg area concurrently with the training sessions. We analyzed electroencephalography (EEG) and balance ability during balance perturbations and changes in cortico-spinal excitability at rest. Balance perturbations were applied by translating the standing surface forward and backward. An acoustic signal was given two seconds before perturbation in an additional condition to reveal the effect of perturbation anticipation on reactive cortical responses.

**Results:**

The results indicate that balance ability, measured by center of mass (COM) displacement and joint excursions, was improved in forward perturbation across all groups, with the anodal stimulation group showing the largest improvement relative to baseline performance following training. Moreover, the anodal stimulation group showed a significant decrease in alpha band power following forward perturbations compared to baseline values after training. N1 latency was reduced across all participants in both perturbation directions after training. However, only the anodal stimulation group showed a significant reduction in backward perturbations compared to baseline values. While training did not induce any significant change in short-interval intracortical inhibition (SICI) measured by Transcranial Magnetic Stimulation (TMS), it increased intracortical facilitation (ICF) in the right tibialis anterior (TA) muscle across all groups, independent of the stimulation condition.

**Conclusions:**

This study provides evidence that tDCS over the M1 area facilitates balance skill acquisition, possibly by facilitating motor preparation and execution and improving the efficiency of sensorimotor integration processes, as shown by decreased alpha power and N1 latency. These findings may have implications for the potential use of tDCS in improving balance control.

**Supplementary Information:**

The online version contains supplementary material available at 10.1186/s12984-025-01663-3.

## Introduction

Balance control is a motor skill that required for various daily activities, requiring movement of body parts or the whole body. Within this domain, standing balance is pivotal because most daily tasks are performed in an upright position.

The body oscillates naturally in the anteroposterior direction like an inverted pendulum while standing still [1]. Physiological processes such as breathing cause additional measurable oscillations, however the inherently unstable pendulum-like mechanical system is the primary driver of these oscillations. When these natural oscillations lead to instability, reactive responses are triggered, recruiting spinal, subcortical, and cortical circuits depending on the magnitude of balance disturbance [2–6]. Even during motionless standing, these reactive responses require cortical involvement [6], and unpredictable or larger external perturbations necessitate greater and faster cortical processing [7, 8].

The ankle strategy is the default mechanism when it is sufficient to recover balance. Here, the ankle muscles (e.g., tibialis anterior (TA) and soleus (SOL) muscles) maintain balance during motionless stance or after small perturbations. When a perturbation exceeds the threshold the ankle strategy can counteract, the body employs the hip strategy, in which larger hip joint excursions move the COM more rapidly than it is possible with the ankle strategy [9, 10]. Therefore, greater hip motion indicates a larger impact of perturbations, and increased postural instability [9, 11]. Balance training is expected to enhance balance control, decrease COM displacement, and consequently reduce the need for large hip excursions after perturbations.

Humans respond to perturbations in a direction-specific manner [12, 13]. The body can lean further forward than backward because of a greater ankle plantarflexion than dorsiflexion torque and a greater flexion than extension range of motion in the hip joint. Thus, the capacity for balance recovery is greater when balance is disturbed in the backward direction, such as after a forward translation of the supporting surface. In addition to these biomechanical differences, perturbations trigger direction-specific postural responses and muscular patterns produced by distinct neural circuits [4, 13]. Thus, many studies have explored cortical and behavioral balance recovery responses separately for different perturbation directions [8, 11, 13–15].

With respect to cortical mechanisms, the N1 wave of perturbation-related evoked potentials, elicited in the supplementary motor area 90–200 ms after perturbation onset, is a crucial indicator [8, 16, 17]. The amplitude and latency of the N1 wave and the power of its underlying frequency bands, especially the theta band (3–7 Hz), are closely associated with balance perturbations [8, 18–20]. Its amplitude reflects sensorimotor processes with the contribution of cognitive processes related to perturbations [21], and is influenced by perceived threat, predictability, and attention [22, 23]. N1 latency shortens as perturbation intensity increases [20, 24, 25], especially when it is strong enough to require a compensatory step, suggesting more rapid cortical engagement to prepare voluntary responses such as stepping [8]. Additionally, more challenging balance tasks have been found to increase mid-frontal theta and alpha power [18, 26, 27]. On the other hand, alpha band power is involved in motor preparation and execution in responses to balance perturbations [19, 28]. With improved sensory integration and experience gained through balance training, reduced N1 amplitude, latency, and theta and alpha band power are however expected, indicating increased automaticity and reduced cortical involvement, accompanied by a shift toward subcortical control [22, 29].

The primary motor cortex (M1) is most likely involved in balance learning at the beginning of balance training, while other motor centers seem to replace this area for long-term motor memory formation [30]. The increasing role of subcortical centers during balance training is relevant for task automatization of acquired balance skills [31]. Inactivation of M1 in the initial stage of motor training disrupted motor skill acquisition in mice, whereas no significant decline in motor performance was observed when M1 was inactivated in the late training stage [32]. However, disrupting M1 activity using low-frequency repetitive transcranial magnetic stimulation (rTMS) did not interfere with balance skill acquisition following a single training session, but impaired retention after 24 h in another study [33]. Despite an increasing number of studies investigating the involvement of M1 in balance learning, the detailed mechanisms underlying balance skill acquisition remain largely unclear. Therefore, this study focuses on the role of M1 in balance skill acquisition, based on the hypothesis that facilitating M1 activity during balance training may enhance balance learning.

TMS, a non-invasive brain-stimulation technique that generates brief electromagnetic fields to induce electric currents in corticospinal and intracortical neurons [34, 35], can be used to quantify training-induced changes in M1. Intracortical inhibitory circuits are typically assessed using short-interval intracortical inhibition (SICI), which probes GABA_A_-mediated inhibitory networks, while excitatory mechanisms can be evaluated using intracortical facilitation (ICF), which probes glutamatergic facilitatory networks. Motor training typically leads to decreased inhibition (SICI) accompanied by increased facilitation (ICF) [36, 37], as decreased SICI is assumed to facilitate the motor learning process through gating of glutamatergic plasticity [38]. However, training in complex, coordinative skills, including balance training, has been reported to increase SICI [39]. For instance, increased SICI was found after four weeks of balance training but then decreased to baseline after 4 weeks of explosive training [40]. Additionally, expert athletes in coordinative sports, such as badminton, demonstrate higher resting inhibition and facilitation than novices, suggesting that long-term practices in coordinative skills may be associated with enhanced intracortical inhibition and facilitation [41]. Based on these findings, we anticipated that short-term balance training would lead to an increase in SICI, whereas evidence for ICF modulation remains limited and inconsistent [42, 43]. Nevertheless, we expected an increase in ICF, as short-term motor training was anticipated to increase facilitatory processes to induce long-term potentiation (LTP).

A non-invasive brain stimulation tool to facilitate M1 excitability is transcranial direct current stimulation (tDCS), which modulates the resting membrane potential of cortical neurons [44]. Anodal tDCS increases excitability and facilitates neuroplasticity and long-term potentiation, which are essential for skill acquisition and retention [45]. Consequently, anodal tDCS improves motor learning [46–49] depending on montage [50], intensity [51], type of task [33], and task complexity [52].

This study investigated the role of the M1 in acquiring balance skills by enhancing neural processing through anodal tDCS. We hypothesized that short-term perturbation-based balance training would improve balance performance, reduce N1 amplitude and latency as well as theta and alpha band frequency power, and decrease intracortical inhibition while increasing facilitation, with larger improvements resulting from anodal tDCS.

## Methods

### Participants

Thirty-four healthy volunteers participated in this study, divided into three groups: the control group (*n* = 11; 5 females, 6 males; age 25.77 ± 1.6 years; weight 71.1 ± 5.3 kg; height 173.0 ± 2.3 cm), the sham group (*n* = 11; 7 females, 4 males; age 27.33 ± 3.23 years; weight 66.77 ± 4.64 kg; height 169.8 ± 3.16 cm), and the anodal tDCS group (*n* = 12; 7 females, 5 males; age 25.2 ± 1.2 years; weight 71.5 ± 4.89 kg; height 173.1 ± 3.56 cm). A priori power analysis was conducted using G*Power, with the following parameters: an expected effect size of 0.20, an alpha level of 0.05, a statistical power of 80%, three groups, two repeated measurements, a correlation between repeated measures of 0.80, and a nonsphericity correction (ε) of 1. Participants were randomly assigned to one of the experimental groups. This study took place at the Department of Psychology and Neurosciences and the Department of Ergonomics, Leibniz Research Centre for Working Environment and Human Factors, Dortmund, Germany. Thirty-four healthy right-handed and right-footed participants were recruited via online advertising and flyers. Only candidates who met none of the exclusion criteria were eligible to participate.

Potential participants were invited to a screening interview before the experiment started. The “TMS/tDCS screening questionnaire”, adapted from Keel et al. and Rossi et al. [53, 54], and the Modified Physical Activity Readiness Questionnaire (PAR-Q) were used to screen potential participants to determine whether they were suitable to participate [55]. The Edinburgh Handedness Inventory was used to determine handedness of potential participants [56]. The mean handedness score of all participants was 0.99 ± 0.04. Participants were asked the following question to determine their foot preference: “If you would kick a ball at a target, which leg would you use to kick the ball?“ [57]. Potential participants read the participant information sheet detailing information about the study, including the health and safety considerations of all protocols, and were asked to sign an informed consent form. The experimental procedures were conducted in accordance with the Declaration of Helsinki and approved by the ethics committee of the Leibniz Research Centre for Working Environment and Human Factors (IfADo). Participants with a history of any neurological disease, a cardiac pacemaker, deep brain stimulation, metal implants in the head or neck, intracerebral ischemia/bleeding, epilepsy, head injury with a skull fracture or brain tissue lesion, any serious medical condition or psychiatric illness, pregnancy or breast-feeding, alcohol or drug addiction, smoking, aphasia, any legal reason preventing participation, participation in another study within the last four weeks, CNS-acting medication intake, any orthopedic disorder or lack of position sense in the lower limbs, vertigo, surgery at spinal cord level, and participation in any balance training were excluded.

### Inclusion criteria

Healthy adults aged 18–55 years, right-handed and -footed, non-smokers, with no history or presence of neurological or psychiatric disorders or other above-mentioned exclusion criteria were included in this study. As a result of the typical aging process, individuals over the age of 55 have modified responses to postural perturbations relative to younger individuals and were thus not included [23, 58].

### Experimental procedure

**Fig. 1:**
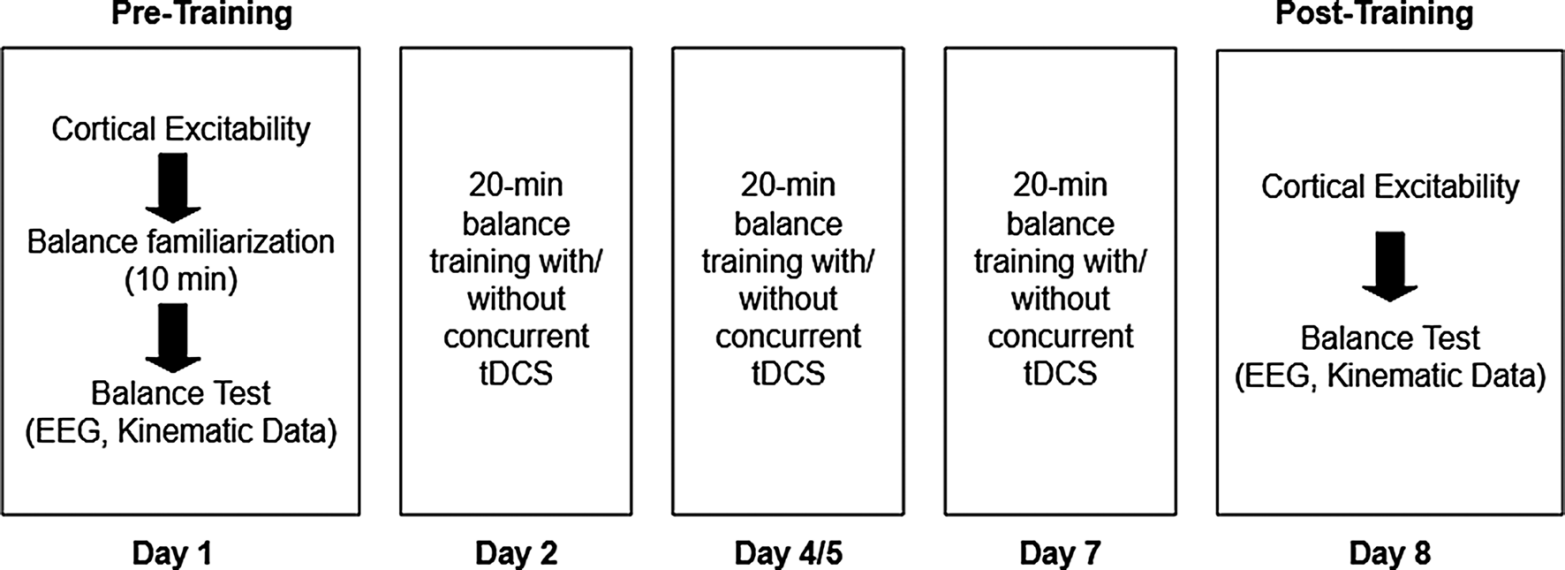
Experimental design. The training was conducted in three sessions within one week, with 24 to 72 h between sessions. In the pre- and post-training sessions, EEG and kinematic data were collected during the balance test, while cortical excitability measures were conducted before it. In the pre-training session, a 10-minutes balance familiarization session was conducted before the balance test. EEG: Electroencephalography, tDCS: transcranial Direct Current Stimulation

Two data collection sessions were conducted before and after one week of balance training. Each assessment session took approximately 6 h, and each training session lasted for about one hour. Participants underwent three training sessions within one week, separated by at least one day. They were assigned to one of three experimental groups: anodal, sham, or no stimulation (control). Participants received one of these stimulations during the training sessions. The design of the study is shown in Fig. 1.

All data used for this project were collected at the laboratories of the Leibniz Research Centre for Working Environment and Human Factors (Dortmund, Germany). Prospective participants received an email with information and related documents about the study and an invitation to the first screening session. In this pre-measurement session, a research staff member provided the potential participants with all required information about the study, explained the procedures, and answered questions about the experiment. Potential participants completed the informed consent forms before they filled in the screening questionnaires. The researchers conducted a TMS measurement with eligible participants to determine whether M1 hotspots with sufficiently large MEPs (0.15–0.5 mV) could be identified for receiving reliable and consistent results across the measurement sessions. Next, participants who were eligible for the study were scheduled for the assessment and training sessions.

The other outcome measures (EEG and kinematic analysis) were obtained during the balance test. These recordings were done in the Gait Real-Time Analysis Interactive Laboratory (GRAIL, Motek Medical, Utrecht, Netherlands). The GRAIL laboratory was equipped with a dual-belt treadmill with two embedded force plates, a VICON motion capture system (VICON, Oxford, United Kingdom) with ten infrared cameras, a 180˚ projection screen placed in front of the treadmill, and three projectors to display a visual scene. The treadmill platform was able to produce surface translations in the sagittal and frontal planes. D-flow software was used to operate the treadmill (Motek Forcelink, Amsterdam, Netherlands).

Participants wore safety harnesses attached to the ceiling to prevent falls and injuries. The participants stood on the platform with their hands on their hips, feet shoulder-width apart, gazing at a cross displayed on the screen, which was adjusted to their eye level. The distance between their first toes and the cross height was measured during the pre-training session to ensure that participants maintained the same base of support area and body orientation during the post-training session.

### Data collection

#### Cortico-spinal excitability measures

Adaptation of inhibitory and excitatory circuits after balance training was assessed via monitoring of motor-evoked potential (MEP) amplitudes. A cone-shaped butterfly coil (D-B80, MagVenture A/S, Farum, Denmark; 95 mm outer diameter, 120º angle) connected to a transcranial magnetic stimulator (MagPro X100, MagVenture A/S, Farum, Denmark) was used for motor cortical excitability measurements, including short-interval intracortical inhibition (SICI) and intra-cortical facilitation (ICF). SICI and ICF were measured during rest before the balance test.

Surface electromyography (EMG) data of TA and SOL muscles on each side were recorded using Ag/AgCl electrodes (Ambu BlueSensor N, Ambu A/S, Ballerup, Denmark). These muscles were selected for their primary roles in controlling balance during anteroposterior perturbations. However, they differ in the strength of their corticospinal projections: the tibialis anterior has stronger corticomotor connections than the soleus muscle [59, 60]. The EMG electrodes were placed in bipolar arrangements with a 2 cm center-to-center distance at the proximal 1/3 of the line between the tip of the fibula and medial malleolus for the tibialis anterior muscle, and at the distal 1/3 of the line between the medial condyle and medial malleolus for the soleus muscle. Before electrode application, the skin where the electrodes were to be placed was shaved, gently abraded with abrasive skin prepping gel (Nuprep Weaver and Company, Aurora, CO, United States), and cleaned with alcohol pads to decrease skin resistance below 2 kOhm. The electrode positioning and skin preparation were conducted in accordance with European recommendations for surface electromyography [61].

The TMS coil was situated over the leg area of the motor cortex, with the handle pointing posterior. The hot spot of the cortical representations of the target muscles, which elicited the largest amplitude of motor evoked potentials (MEP) with medium TMS intensity, was identified in the resting TA and SOL muscles of both hemispheres by moving the coil to various locations. A water-proof marker was used to mark the position of the coil after identifying the hotspot and the locations of EMG electrodes after attaching them to guarantee the exact repositioning of the coil and EMG electrodes in the post-training session. These marks were checked and renewed, when necessary, before each training session.

The resting motor threshold (rMT) was then determined by the TMS-Motor-Threshold-Assessment Tool (MTAT 2.0) [62] using the “without a priori information” method. To determine rMT, feedback was provided to the software by selecting “yes” if the MEP amplitude exceeded 50 µV, and “no” if it did not. The program determines the next TMS intensity to be given depending on the provided input. After a sufficient number of stimuli, the software displays the rMT within the 95% confidence interval.

A paired-pulse TMS protocol (two single TMS pulses separated by 2 ms for SICI and 10 ms for ICF) was applied to assess intracortical excitability. This was accomplished by providing a subthreshold TMS conditioning stimulus pulse delivered prior to the test stimulus. We applied a conditioning stimulus intensity of 80% rMT [63]. Providing the conditioning stimulus before the test stimulus changes the amplitude of the MEP in response to the test stimulus, depending on the interstimulus interval [35]. The test stimulus intensity, which elicited MEPs within the 0.15–0.5 mV range, was determined. This range was determined after pilot testing to identify the most proper MEP amplitude for the target muscles. MEP amplitudes in response to the test stimulus, rMTs, and test stimulus intensities for each muscle, group, and session are shown in Table 1.

Fifteen MEPs for SICI, ICF, and single pulse TMS were recorded during assessment. The largest and smallest MEP for SICI, ICF, and single pulse stimulation were removed before statistical analyses to eliminate outliers, as these might result in skewed average values [64]. Then the means of thirteen MEPs per condition were calculated. SICI and ICF were quantified for each muscle by dividing the MEP amplitude in response to the test stimulus in the double pulse condition by the MEP amplitude in the single pulse condition. Therefore, smaller SICI values indicate stronger intracortical inhibition, whereas a higher ICF value reflects enhanced intracortical facilitation. SICI and ICF for each muscle (TA and SOL on both sides) were calculated as inputs into the statistical analysis.

**Table 1:** MEP amplitudes, resting motor thresholds (rMTs), and test stimulus intensities for each muscle, group, and session

		Control (mean ± SD (min-max))		Sham (mean ± SD (min-max))		Anodal (mean ± SD (min-max))	
		Pre-training	Post-training	Pre-training	Post-training	Pre-training	Post-training
MEP (µV)	Right TA	278.12 ± 96.85 (160.11–455.04)	247.98 ± 89.84 (175.35–427.04)	250.35 ± 52.05 (169.87–329.59)	236.90 ± 74.33 (140.84–381.25)	260.33 ± 69.17 (175.78–392.58)	258.19 ± 58.16 (183.11–378.93)
	Right SOL	251.69 ± 100.48 (152.64–483.67)	215.87 ± 70.42 (130.12–343.26)	227.87 ± 71.67 (149.59–358.45)	211.88 ± 52.05 (121.29–308.61)	218.05 ± 46.64 (151.88–315.64)	218.37 ± 60.57 (149.01–325.88)
	Left TA	283.81 ± 77.87 (149.66–416.98)	281.62 ± 78.51 (171.20–385.55)	267.48 ± 90.00 (192.40–505.27)	290.96 ± 108.16 (198.51–489.69)	293.81 ± 66.99 (194.75–404.39)	284.59 ± 77.84 (199.28–460.78)
	Left SOL	277.69 ± 104.95 (149.69–454.10)	243.28 ± 84.11 (137.62–400.77)	218.27 ± 56.40 (156.74–329.03)	211.84 ± 51.70 (115.66–298.26)	228.34 ± 56.35 (155.03–331.50)	236.04 ± 58.26 (157.66–354.94)
rMT (%MSO)	Right TA	37.18 ± 4.09	38.82 ± 3.52	41.73 ± 5.24	43.82 ± 6.19	38.33 ± 6.27	39.92 ± 6.86
	Right SOL	41.36 ± 5.87	41.27 ± 4.24	41.91 ± 5.22	43.91 ± 5.13	40.58 ± 6.43	41.67 ± 7.40
	Left TA	38.09 ± 4.99	39.36 ± 5.41	41.18 ± 6.06	41.36 ± 5.48	39.42 ± 4.80	40.42 ± 3.55
	Left SOL	38.91 ± 7.05	40.27 ± 7.56	41.27 ± 4.92	44.00 ± 5.90	41.17 ± 4.63	41.50 ± 5.37
Test Stimulus Intensity (%MSO)	Right TA	58.09 ± 6.04	61.73 ± 7.18	62.64 ± 9.30	64.36 ± 8.74	58.50 ± 10.30	60.17 ± 8.38
	Right SOL	68.55 ± 9.19	72.82 ± 8.80	70.27 ± 8.27	71.82 ± 7.95	65.92 ± 10.02	70.50 ± 11.23
	Left TA	59.00 ± 9.57	61.64 ± 9.71	66.64 ± 6.22	69.18 ± 6.49	61.58 ± 8.98	65.50 ± 9.25
	Left SOL	64.18 ± 12.80	69.91 ± 13.40	71.64 ± 7.67	73.91 ± 7.65	68.42 ± 6.49	70.67 ± 7.64

MEP: motor evoked potential, %MSO: percentage of maximum stimulator output, SD: standard deviation

#### Electroencephalography

EEG data were collected using LiveAmp (Brain Products GmbH, Germany) during the perturbation test with 28 Ag/AgCl electrodes (Fp1, Fp2, F3, Fz, F4, FC5, FC1, FCz, FC2, FC6, T7, C3, C1, Cz, C2, C4, T8, TP9, CP5, CP1, CPz, CP2, CP6, P3, Pz, P4, O1, O2). TP10 was used as the online reference electrode, and AFz was used as the ground electrode. A cap size of 54 to 60 cm was selected based on the participant’s head circumference. After fitting the cap, the electrodes were filled with electrolyte gel to reach an impedance below 10 kΩ. Participants wore a belt carrying the LiveAmp amplifiers (LiveAmp 64, Brain Products GmbH, Germany), battery, sensor & Trigger Extension (LiveAmp 64, Brain Products GmbH, Germany). Electrode cables were arranged to prevent crossing, and movement of both the cables and belt-mounted hardware relative to the body was minimized. This allowed these to move together with, and not independent from participants, reducing EEG artifacts.

EEG data were recorded at a sampling rate of 1000 Hz directly onto a micro-SD card that was inserted into both connected amplifiers. The online data stream from the amplifiers’ Bluetooth connection was monitored via Brainvision Recorder software (Brain Products GmbH). When the perturbation started, an external trigger interface (Phidget Interface Kit, Phidgets Inc., Canada) generated a trigger signal to synchronize perturbation onset with EEG recordings. The trigger signals and EEG data were recorded simultaneously with a wireless Brain Products trigger (Gilching, Germany). After completing the recordings, the EEG data were transferred from the SD card to a PC via LiveAmp File Converter software (Brain Products GmbH). The files containing kinematic variables and belt movements were also transferred for analysis.

#### Kinematic measurements

**Fig. 2:**
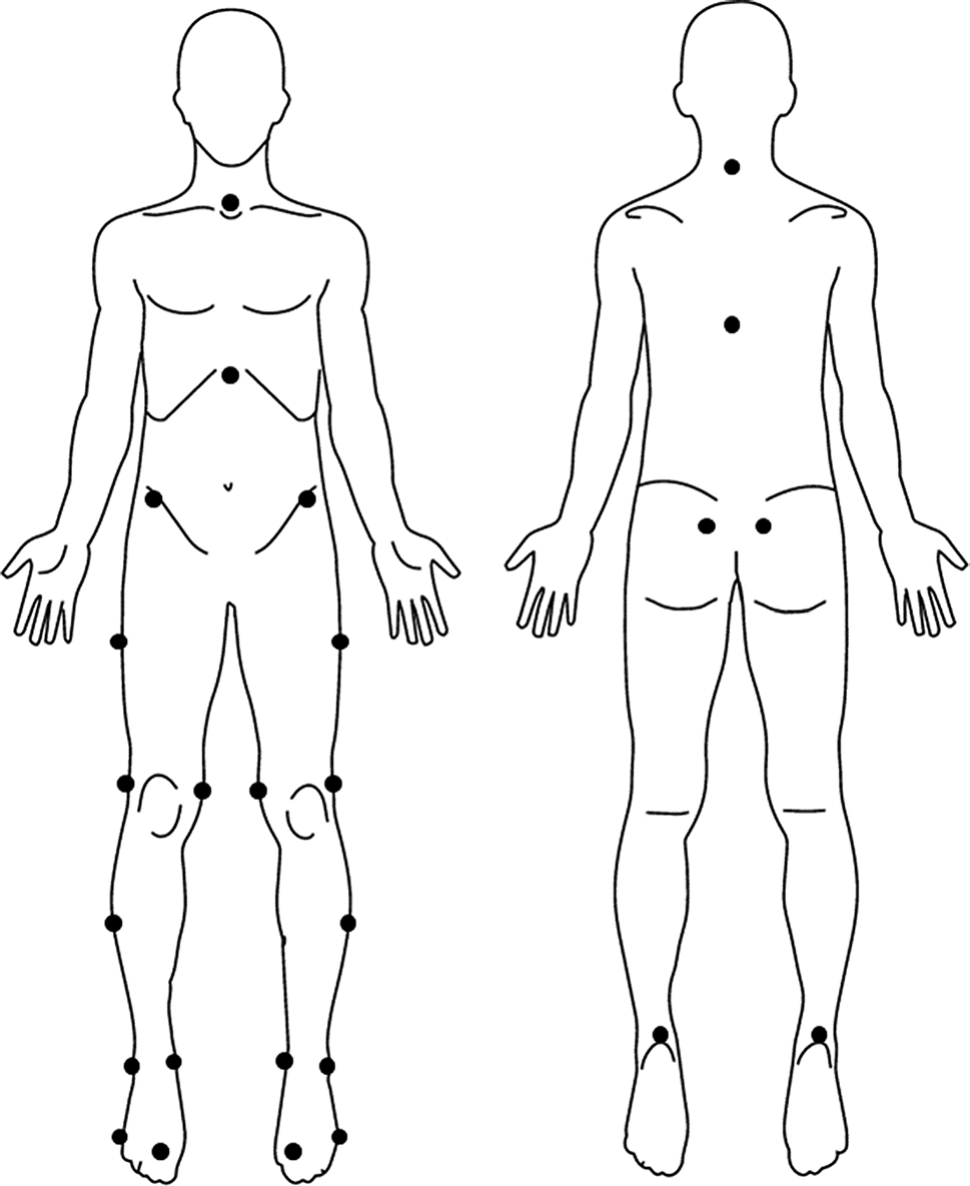
The locations of reflective markers based on the trunk model of the Human Body Model (HBM)

The GRAIL was used to capture center of mass (COM) movement data using a 10-camera Vicon Nexus 3D motion analysis system at a sampling rate of 100 Hz. Reflective markers were placed on the body according to the Human Body Model (HBM) with the trunk model. The markers were located bilaterally at the base of the second and fifth toes, medial and lateral malleolus, heel, medial and lateral epicondyles, midline of the shank and thigh, the spina iliaca anterior superior and spina iliaca posterior superior; and on T10, C7, jugular notch and xiphoid process of the sternum (Fig. 2). An online low-pass filter with a cut-off frequency of 6 Hz was applied using D-flow software (Motek Forcelink, Amsterdam, Netherlands). The D-Flow software calculated and provided relative joint angles between adjacent body segments and center of mass (COM) movements. Based on these data, peak COM displacements and joint excursions of the ankle, knee, and hip joints after perturbations were calculated.

#### Perturbation training protocol

The training was completed within one week and comprised three sessions, each separated by at least one day [29, 65]. Each training session consisted of ten blocks, each containing 12 perturbations delivered on a treadmill at intervals of 3 to 6 s, with each block lasting approximately 93 s. There was a 30-s break after each block. Participants were exposed to three perturbation conditions in each direction (forward and backward): 2 cm with a peak velocity of 0.17 m/s, 4 cm with a peak velocity of 0.35 m/s, and 6 cm with a peak velocity of 0.54 m/s. The first two sessions were conducted during the feet shoulder-width apart position, and the last session was done in the feet together position to increase difficulty. Standing balance becomes more challenging when the feet are placed together because the base of support is narrower, which reduces stability and demands greater control to maintain balance [66, 67].

**Fig. 3:**
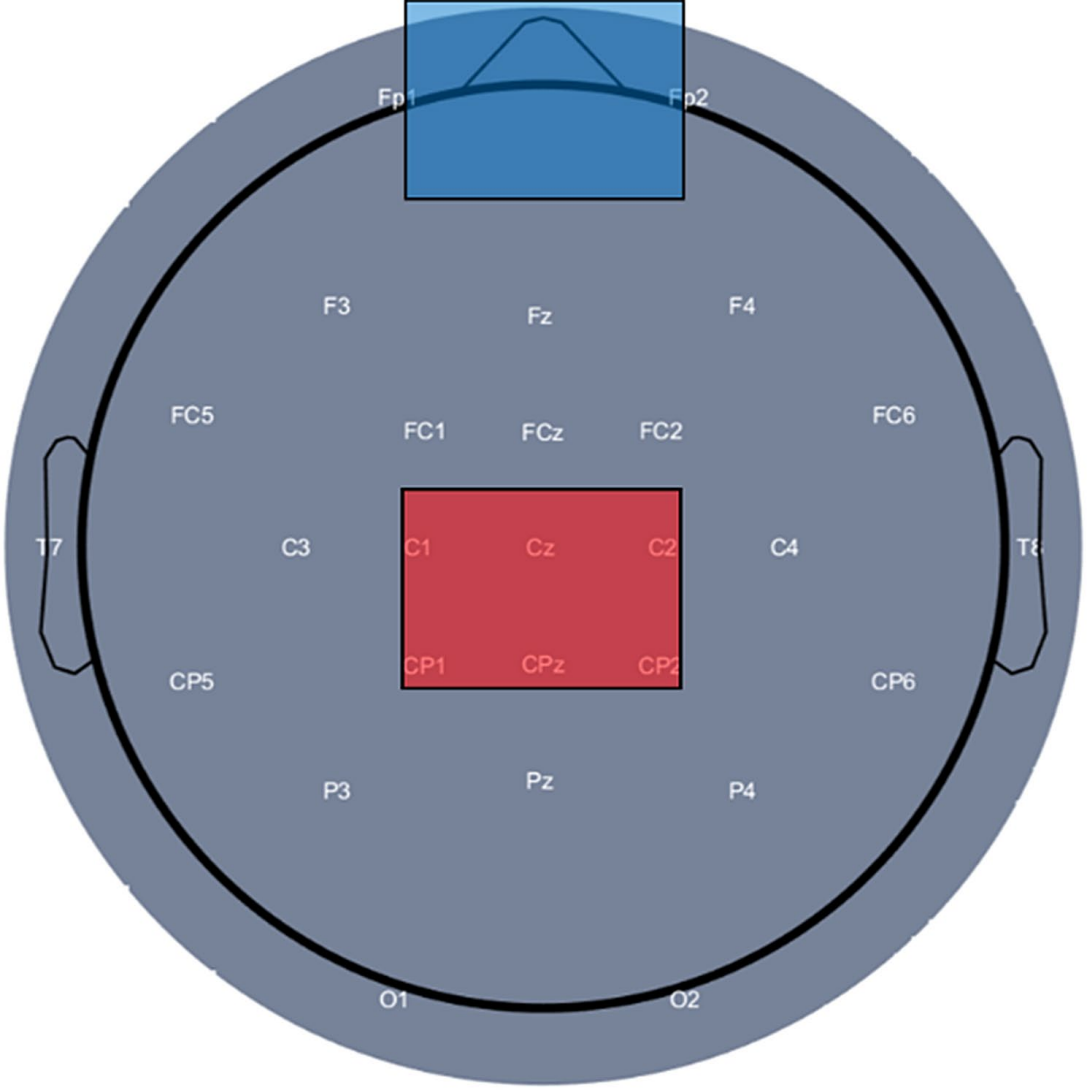
Montage of the tDCS electrodes. The target electrode (red) was placed 1 cm posterior to the Cz, and the return electrode (blue) was located over the Fpz electrode position

As part of the training, participants were given transcranial direct current stimulation (tDCS) according to their assigned group (anodal, sham, or no stimulation). Stimulation was applied concurrently with balance training for 20 min [48, 68, 69] with a Starstim stimulator (NeuroElectrics, Spain) through two sponge electrodes (target and return electrode area: 35cm^2^). The center of the target electrode was placed 1 cm posterior to the vertex (Cz) on the mid-sagittal line to cover both leg motor cortices, and the return electrode was placed with the center over Fpz [49, 50] (Fig. 3). This placement reliably covered all target muscles in each participant, as verified by TMS-defined motor hotspot locations.

A delivered current density of 0.085 mA/cm^2^ was obtained by setting the current at 3 mA. This current density was selected based on a previous study that investigated cortical excitability following tDCS at intensities ranging from 1 to 3 mA. The findings suggested that higher stimulation intensities modulate larger and deeper cortical regions, which is relevant for targeting the lower extremity motor area located in the interhemispheric fissure [70]. Another study demonstrated that a current density of 0.08 mA/cm² significantly increased cortical excitability, whereas a lower current density of 0.04 mA/cm² did not produce a significant change [71]. These findings support that the higher current densities are effective in modulating cortical excitability, particularly when targeting surface-distant motor areas. Current was initially increased in 0.1 mA increments for 30 s until the target current intensity was reached. The anodal stimulation group received constant current during training. Current was ramped up to 3 mA and down to 0 mA within 30 s in the sham group [72, 73]. A topical anesthetic cream (EMLA, 2.5% lidocaine & 2.5% prilocaine) was applied over the stimulation site before each training/stimulation session to improve blinding by reducing the somatosensory perception of stimulation. The control group received no stimulation.

#### Balance test protocol

GRAIL and the Motek Forcelink were used to produce perturbations and collect data on the center of mass (COM) and joint excursions. In the first assessment session, participants participated in a 10-minute familiarization session once they wore the EEG cap, the belt equipped with the data collection devices, and the harness [74].

During the balance test, participants were exposed to surface translations with an amplitude of 6 cm applied in both forward and backward directions in different trials. An acoustic signal was randomly presented two seconds before half of the perturbations to assess the effects of perturbation predictability on cortical responses. This resulted in four experimental conditions, defined by the combination of perturbation direction (forward/backward) and presence or absence of the acoustic signal (no sound/sound). Perturbation conditions were applied in a randomized order, with intervals between trials varying randomly from three to six seconds after the platform returned to its original position.

Fifteen perturbation stimuli were applied for each condition in an assessment session. Perturbations were delivered at a peak velocity of 0.54 m/s and remained in the final position for 2 s before returning to the initial position at a peak velocity of 0.047 m/s. During the familiarization session, participants completed the same balance test protocol for 10 min.

### Data processing

#### EEG analysis

The data were analyzed using MATLAB (version 2023b, Massachusetts, The MathWorks Inc.) and the EEGLab toolbox [75]. An online anti-aliasing filter of 499 Hz was used during EEG data collection. The data were pre-processed using a combination of custom MATLAB scripts and EEGLAB functions. Pre-processing steps included a high-pass filter using a 4th order, zero-phase Butterworth filter with a 1 Hz cutoff, followed by downsampling to 250 Hz to improve line noise removal and to facilitate independent component analysis (ICA) decomposition, identify problematic channels using the bemobil_detect_bad_channel function and re-referencing using the bemobil_avref function to employ the full rank average reference. The data were processed with zapline plus to remove the noisy frequency components. Then, the data were epoched between − 2000 and + 1000 ms relative to perturbation onset before ICA post-processing was run to reject non-brain-originated components. Further analysis was focused on the Cz electrode over M1 and SMA. Finally, event-related spectral perturbation (ERSP) analysis was conducted to calculate the power change relative to baseline window 1000 to 200 ms before perturbation onset in different frequency bands (theta (3–7 Hz) and alpha (8–13 Hz) in the relevant time bin (90–250 ms)). This time range of interest was determined according to previous studies showing that cortical involvement starts at ~ 90 ms, and stepping responses start at around 250 ms after perturbation onset [2, 10, 76]. The N1 potential was calculated as the most negative value of the event-related potential (ERP) between 90 and 200 ms after perturbation onset, relative to the baseline described above. N1 amplitude, N1 latency, and theta and alpha band power for each condition were calculated as inputs into the statistical analysis.

Only feet-in-place responses were included for further analysis. This type of response occurs when the perturbation impact is sufficiently small allowing participants to maintain balance without requiring a compensatory step. We collected 510 trials per perturbation condition (34 participants × 15 trials). In forward perturbations, 47 and 53 stepping responses occurred in the no-sound and sound conditions, respectively. In backward perturbations, 6 and 9 stepping responses were observed in the no-sound and sound conditions, respectively.

#### Kinematic analysis

The kinematic data were analyzed using a custom-made MATLAB script (version 2023b, Massachusetts, The MathWorks Inc.). COM displacements in the horizontal plane relative to the ankle joint and joint angles (ankle, knee, and hip joint on each side) were calculated over a 1200 ms epoch, from − 200 ms to + 1000 ms relative to perturbation onset. COM analysis was limited to the anteroposterior direction because the surface translation and associated COM displacements occur in this axis [77, 78].

As part of the pre-processing procedures, the average COM position and each joint angle within the baseline window from − 1000 to -200 ms relative to the perturbation onset, consistent with the EEG analysis, were calculated [27], and subtracted from each respective variable (COM or joint angle) to normalize the data. Subsequently, post-processing was performed by averaging the COM and joint angle epochs for each perturbation condition and participant. The absolute peak COM displacement and joint excursions after perturbation onset were calculated. Peak COM displacement and peak hip, peak knee, and peak ankle excursions of both sides for each condition (forward + sound, backward + sound, forward + no-sound, backward + no-sound) were calculated as inputs into the statistical analysis.

#### Statistical analysis

A linear mixed-effects model was fitted using the “lmer” function from the “lme4” package in R version 4.3.3 (R Foundation for Statistical Computing) [79]. We treated time (pre, post), group (control, sham, anodal), and sound (no sound, sound) as fixed effects and participants as random effects in the model for EEG and kinematic analysis. Time, group and muscle (Right TA and SOL, left TA and SOL) factors were considered as fixed effects and participants as random effects in the intracortical excitability measures. ANOVA tables were obtained using the “lmerTest” package, which applies type III analysis of variance using Satterthwaite’s approximation for degrees of freedom [80]. Normality of the residuals was assessed through visual inspection of Q-Q plots.

When main and/or interaction effects of the ANOVAs were significant, post-hoc tests were conducted by calculating estimated marginal means using the “emmeans” package with Tukey adjustments. This analysis used the “emmeans” function to obtain “estimated marginal means” by averaging over time, sound, and group within the linear mixed-effect model. Following this, the “contrasts” function was employed to perform pairwise comparisons between these estimated means.

Effect sizes were calculated as partial eta-squared (η_p_^2^) using the “eta_square” function in the “effectsize” package in R for all ANOVA main and interaction effects. Effect sizes were categorized as small (≥ 0.01 and < 0.06), medium (≥ 0.06 and < 0.14), and large (≥ 0.14). Effect sizes for post-hoc comparisons were computed using the “eff_size” function in the “emmeans” package. These effect sizes were expressed by Cohen’s d and categorized as small (d < 0.50), medium (d = 0.5–0.8), or large effects (d > 0.8). The post-hoc comparisons can be found in the supplementary material. The level of significance was < 0.05.

## Results

### Peak COM displacement

The ANOVA revealed a significant main effect of sound in both forward (*p* = 0.00422, η_p_^2^ = 0.09) and backward perturbations (*p* = 0.00053, η_p_^2^ = 0.09) with a medium effect size. Moreover, a significant interaction between time and group factors was revealed in forward perturbations (*p* = 0.0497, η_p_^2^ = 0.06), also with a medium effect size. No other significant main or interaction effects emerged (*p* = 0.0586–0.861). Detailed statistics for the mixed-ANOVA are shown in supplementary material, Table 1.

**Fig. 4:**
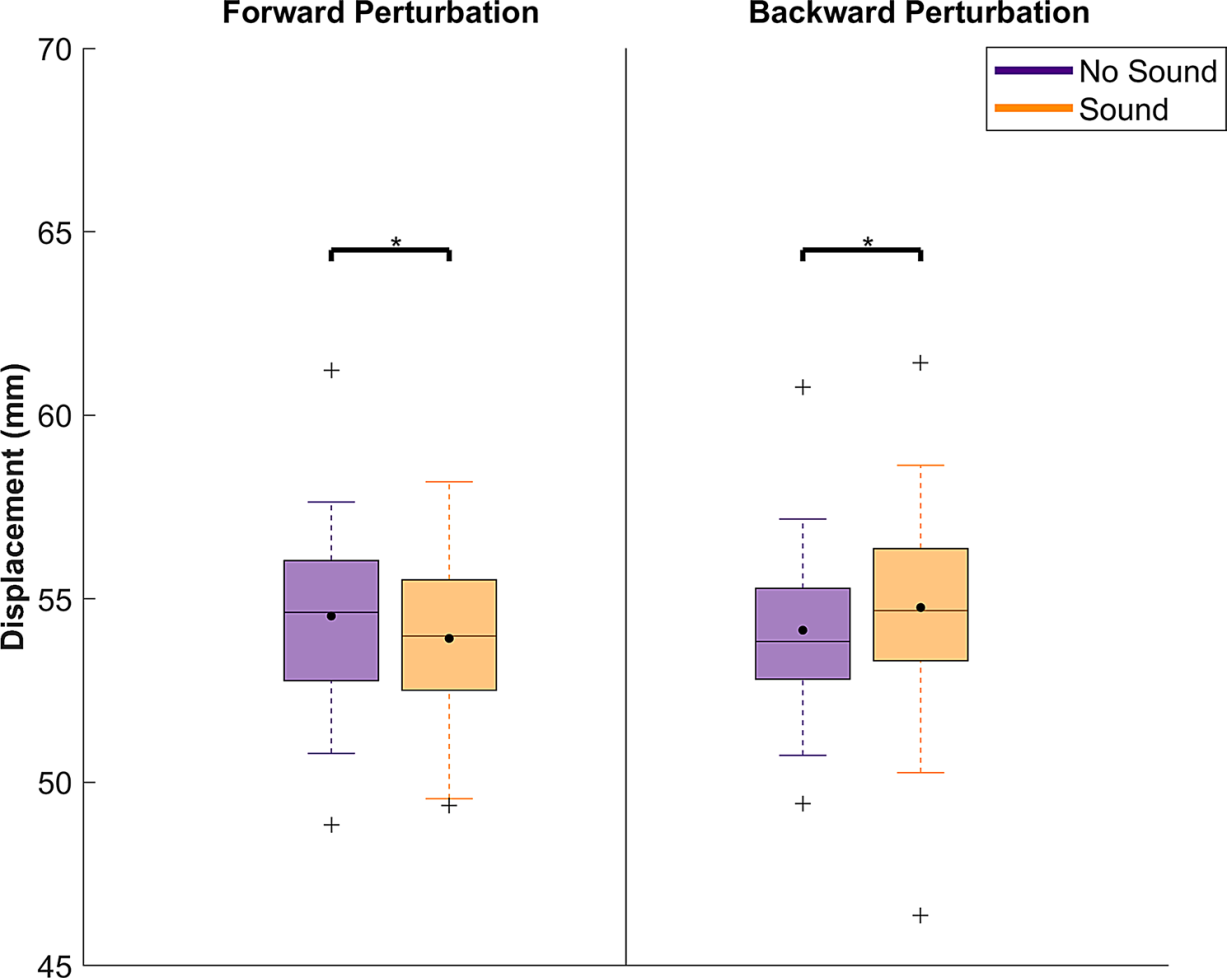
Peak COM displacement comparisons between sound conditions. The data were averaged over time, as well as by group factors. Each box shows the interquartile range (25th-75th percentiles), and the horizontal line in each box indicates the median, while the black dot indicates the mean. Outliers are indicated with a “+” and whiskers represent the range of data without outliers. Asterisks indicate significant differences between “no sound” and “sound” conditions

**Fig. 5:**
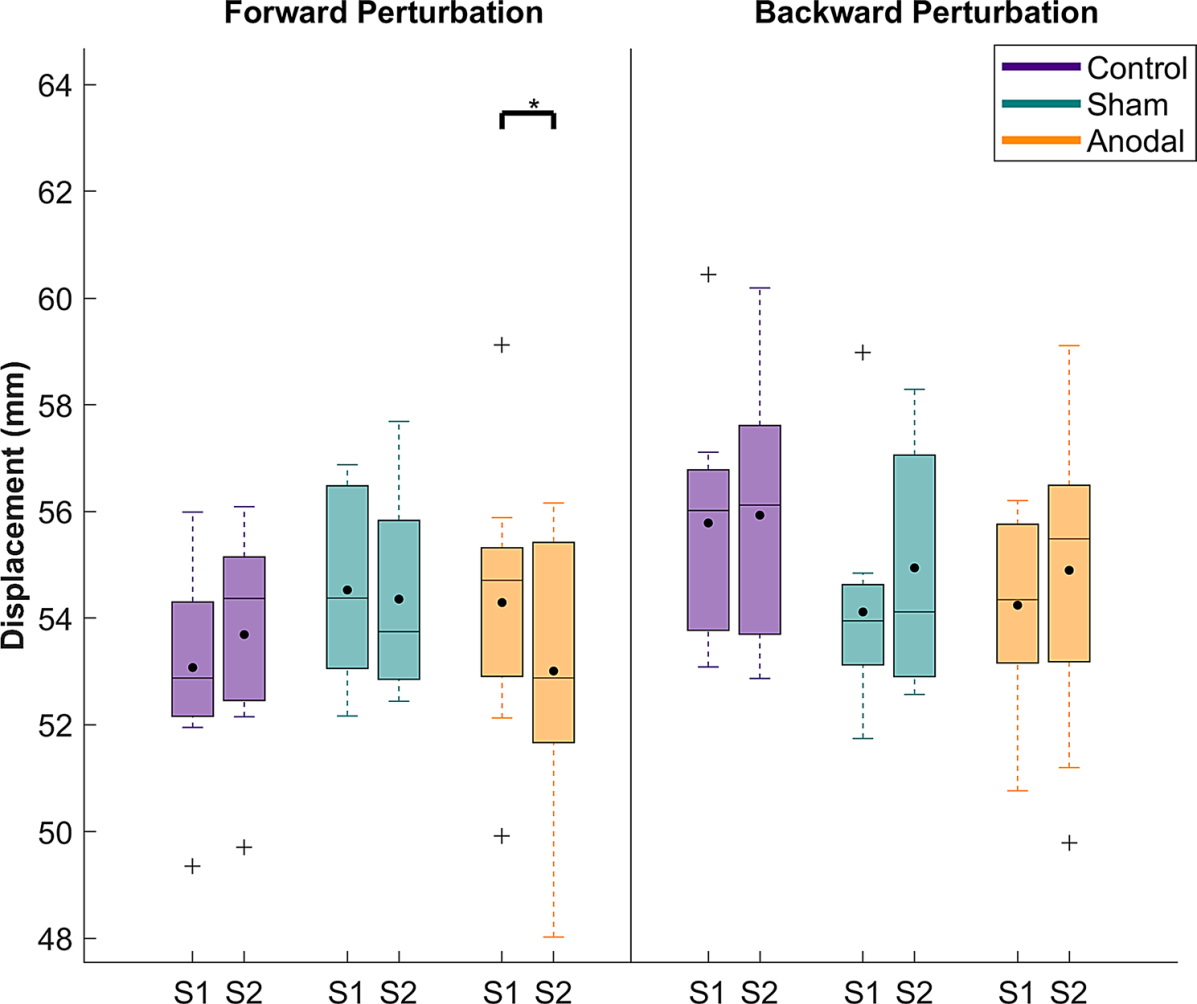
Peak COM displacements for each group and each perturbation direction (averaged over the sound conditions). Each box shows the interquartile range (25th-75th percentiles), and the horizontal line in each box indicates the median, while the black dot indicates the mean. Outliers are indicated with a “+” and whiskers represent the range of data without outliers. Asterisks indicate significant differences before and after training. S1 = Pre-training, S2 = Post-training

Post-hoc comparisons revealed that the sound factor affected COM displacement differently for forward and backward perturbation (Fig. 4). Specifically, for forward perturbation, sound conditions caused a significantly smaller COM displacement than the no sound condition (*p* = 0.004, d = 0.51, supplementary material Table 6). In contrast, for backward perturbations, sound conditions led to a larger COM displacement compared to no sound conditions (*p* = 0.0005, d = 1.04, supplementary material Table 9). For the time x group interaction in the forward perturbation, the post-hoc comparisons showed that only the anodal stimulation group showed significantly decreased peak COM displacements following training, as shown in Fig. 5 (*p* = 0.02, d = 0.69, supplementary material Table 7).

### Peak joint excursions

A significant main effect of time was observed in the right hip (*p* = 0.016, η_p_^2^ = 0.07), right knee (*p* = 0.00056, η_p_^2^ = 0.14), and right ankle (*p* = 0.023, η_p_^2^ = 0.06) joints in forward perturbations and in right and left knee joints (*p* = 0.00045, η_p_^2^ = 0.14 and *p* < 0.0001, η_p_^2^ = 0.18, respectively) in backward perturbations, with medium to large effect sizes. A significant time x group interaction was observed for the right and left hip joints (*p* = 0.00916, η_p_^2^ = 0.11, *p* = 0.0168, η_p_^2^ = 0.1, respectively), as well as the left ankle joint (0.00154, η_p_^2^ = 0.15), in the forward perturbations, with medium to large effect sizes. All other main or interaction effects were not significant (*p* = 0.0652–0.997). Detailed statistics of the mixed-model ANOVA are shown in supplementary material, Table 2.

For the main effect of time in the forward perturbation condition, the post-hoc comparisons showed that joint excursions in the right hip (*p* = 0.016, d = 0.45, supplementary material Table 10), right knee (*p* = 0.0006, d = 0.66, supplementary material Table 15) and right ankle joints (*p* = 0.023, d = 0.43, supplementary material Table 16) were reduced compared to before training across all groups. For the main effect of time in the backward perturbation, on the other hand, right and left knee joint excursions were significantly increased across all groups following training (*p* = 0.0005, d = 0.68 and *p* = 0.0001, d = 0.76, respectively, supplementary material Tables 19 and 20).

For the time x group interaction in the forward perturbation condition, the post hoc analysis revealed furthermore that only the anodal stimulation group showed a significant bilateral decrease of hip joint excursion following training compared to the pre-training session (*p* = 0.0001, d = 1.25, and *p* = 0.0061, d = 0.87, right and left side, respectively, supplementary material Tables 11 and 13), without, however, significant differences between intervention groups.

**Fig. 6:**
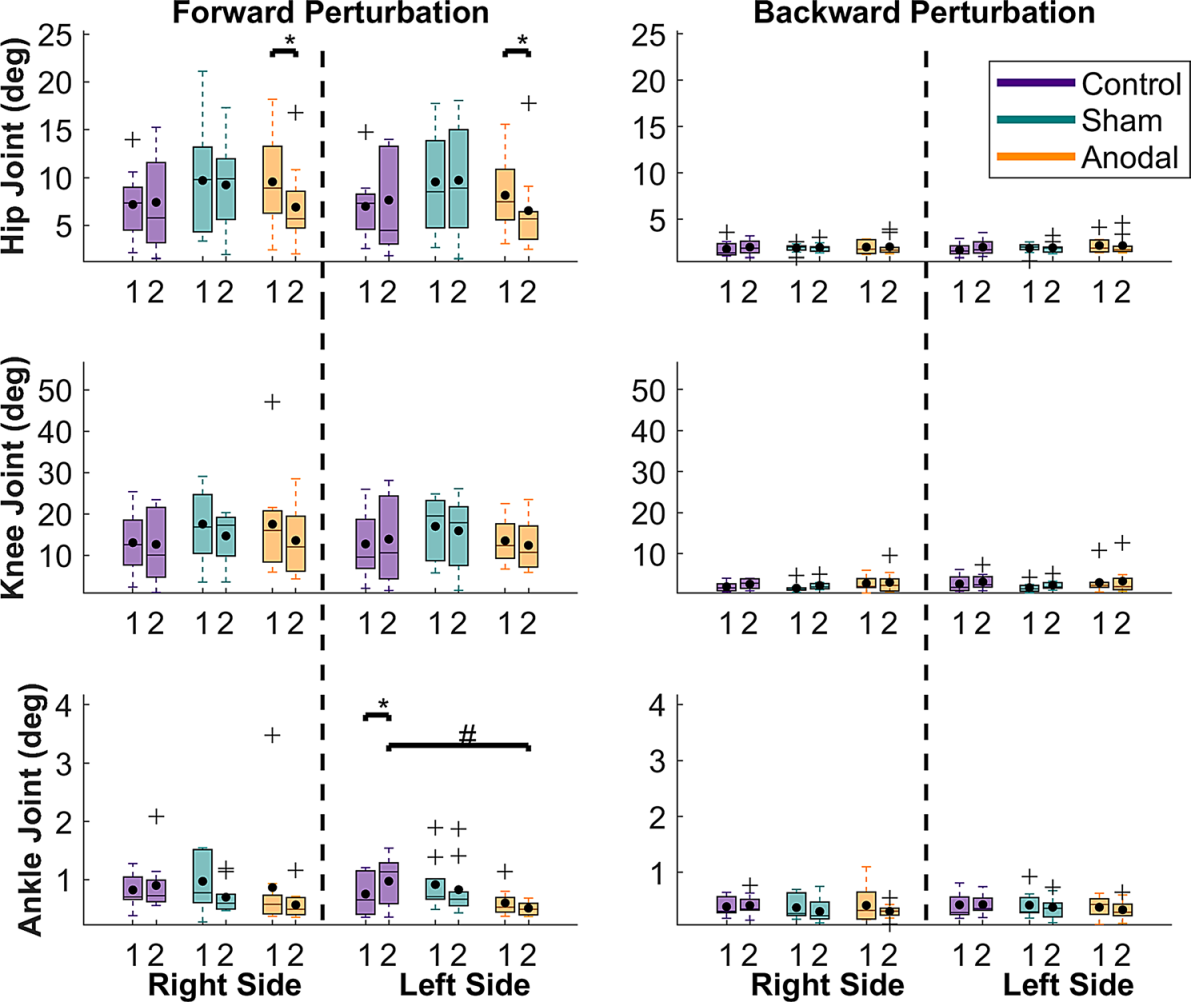
Peak joint excursions in the period of interest after perturbation onset (100–250 ms) before and after training for lower extremity joints in each group and each perturbation condition. Each box shows the interquartile range (25th-75th percentiles), and the horizontal line in each box indicates the median, while the black dot indicates the mean. Outliers are indicated with a “+” and whiskers represent the range of data without outliers. Asterisks indicate significant differences before and after training, while hashtags demonstrate significant differences between groups. Pre- and Post-Training are labeled as “1” and “2” in the figures, respectively

For the time x group interaction in the forward perturbation, left ankle joint excursions increased significantly only in the control group following the training.

(*p* = 0.0015, d = 1.1, supplementary material Table 17) and it was significantly larger than the anodal stimulation group in the post-training session (*p* = 0.0223, d = 1.35, supplementary material Table 18). The joint excursions before and after training for each group for each joint are illustrated in Fig. 6.

### N1 responses

According to the results from the mixed-model ANOVA, the N1 amplitude showed significant main effects of Time (*p* = 0.0038, η_p_^2^ = 0.09), Sound (*p* = 0.00102, η_p_^2^ = 0.11), and Group (*p* = 0.0417, η_p_^2^ = 0.19) for backward perturbation, with medium to large effect sizes. Additionally, a significant main effect of time was observed for N1 latency in both forward and backward perturbations (*p* < 0.0001, η_p_^2^ = 0.29 and *p* < 0.0001, η_p_^2^ = 0.16, respectively), with a large effect size. Furthermore, a significant time x group interaction for N1 latency in backward perturbations was found (*p* = 0.0308, η_p_^2^ = 0.07), with medium effect size. No additional main or interaction effects were observed (*p* = 0.0626–0.984). Detailed statistics of the mixed-model ANOVAs are shown in supplementary material, Table 3.

For the main effect of the time in the backward perturbation condition, post-hoc comparisons showed that the perturbation-based balance training significantly reduced the N1 amplitude across all groups (*p* = 0.0038, d = 0.52, supplementary material Table 21). For the main effect of sound in the backward perturbation condition, the post-hoc analysis revealed that the N1 amplitude was significantly smaller in the sound conditions compared to the no sound conditions (*p* = 0.001, d = 0.59, supplementary material Table 22). For the group main effect in the backward perturbation, the post-hoc analysis showed that the N1 amplitude in the sham stimulation group was significantly larger compared to the control group (*p* = 0.0412, d = 3.36, supplementary material Table 23).

**Fig. 7:**
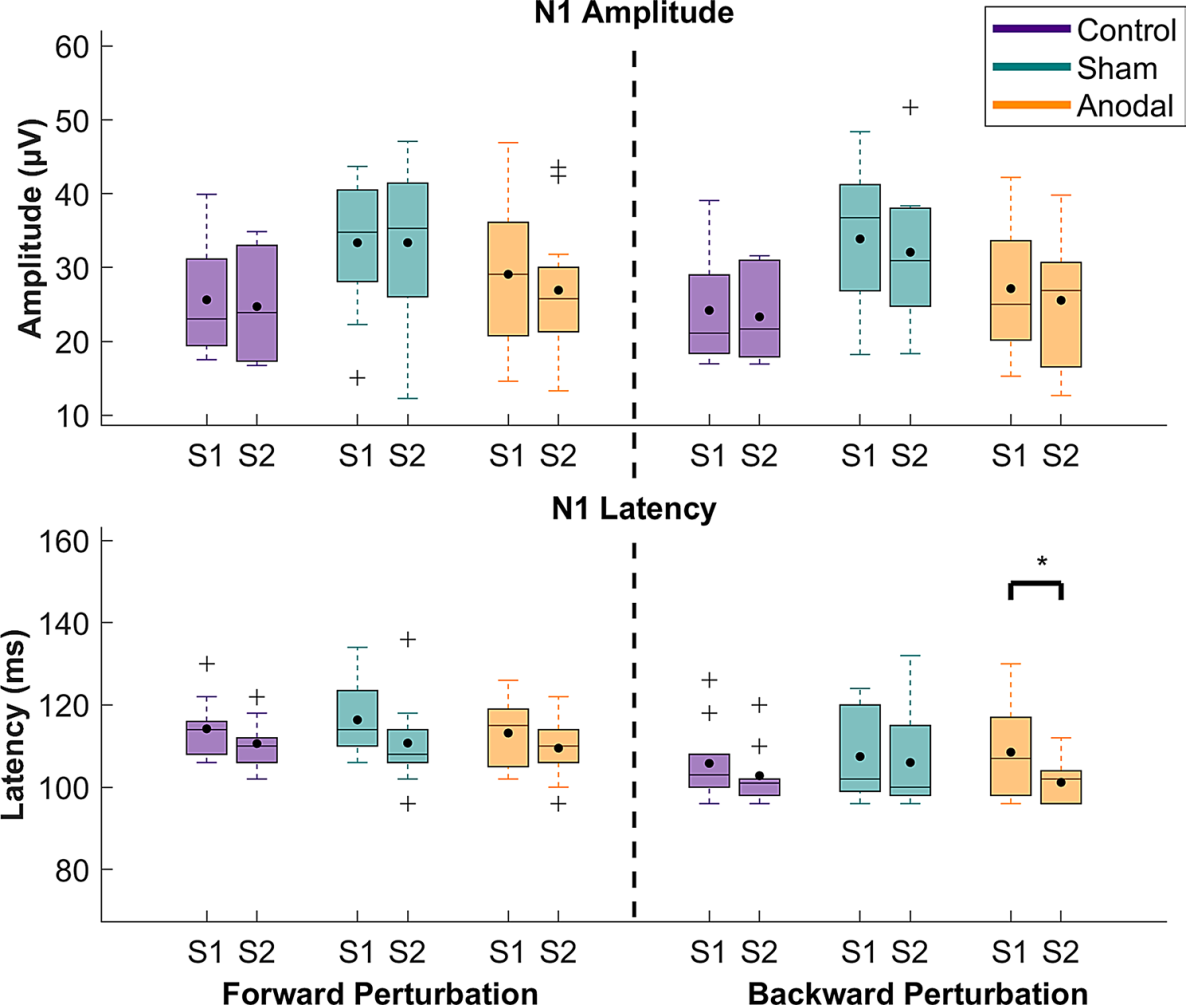
N1 amplitudes and latencies for each group and each perturbation direction (averaged over the sound factor) before and after training. The amplitude data were standardized via baseline correction with the baseline window within a range of 1000 to -200 ms, prior to the perturbation onset. Each box shows the interquartile range (25th-75th percentiles), and the horizontal line in each box indicates the median, while the black dot indicates the mean. Outliers are indicated with a “+” and whiskers represent the data range without outliers. Asterisks indicate significant differences before and after training. S1 = Pre-training 1, S2 = Post-training

For the main effect of time in both forward and backward perturbation conditions, perturbation-based balance training caused a significant reduction of N1 latency across all groups (*p* < 0.0001, d = 1.07 and *p* = 0.0001, d = 0.72, respectively, supplementary material Tables 24 and 25). For the time x group interaction, only the anodal stimulation group demonstrated a significant N1 latency reduction following training (*p* < 0.0001, d = 1.35, supplementary material Table 26). The changes in each group for both N1 amplitude and latency are illustrated in Fig. 7.

### Average theta and alpha band power (Cz)

A significant main effect of time was found for theta power in forward perturbations (*p* < 0.0001, η_p_^2^ = 0.18). We also found a significant main effect of sound for theta power in both forward and backward perturbation conditions (*p* = 0.0496, η_p_^2^ = 0.04, *p* = 0.00354, η_p_^2^ = 0.09, respectively), with small and medium effect sizes. The ANOVA results furthermore revealed a significant effect of sound on alpha power only for backward perturbation (*p* = 0.0032, η_p_^2^ = 0.09), with a medium effect size. A statistically significant interaction between time and group factors was observed for theta band power in the backward perturbation condition (*p* = 0.007, η_p_^2^ = 0.1), with a medium effect size. In contrast, for alpha band power, a significant interaction between time and group in the forward perturbation direction was shown (*p* = 0.0395, η_p_^2^ = 0.07), with a medium effect size. No other main or interaction effects were significant (*p* = 0.0885–0.841). Detailed statistics for the mixed-model ANOVA are shown in supplementary material, Table 4.

For the main effect of time in the forward perturbation condition, the post-hoc comparisons revealed that theta power was significantly reduced following perturbation-based balance training across all groups (*p* < 0.0001, d = 0.78, supplementary material Table 28). The post-hoc comparisons showed that the acoustic signal significantly decreased theta power compared to the no sound conditions for the main effect of sound in both forward and backward perturbations (*p* = 0.0496, d = 0.3 and *p* = 0.0035, d = 0.52, respectively, supplementary material Tables 29 and 30). Alpha power showed a significant reduction in the presence of the acoustic signal in backward perturbations, but not in forward perturbations (*p* = 0.0032, d = 0.53, supplementary material Table 35).

**Fig. 8:**
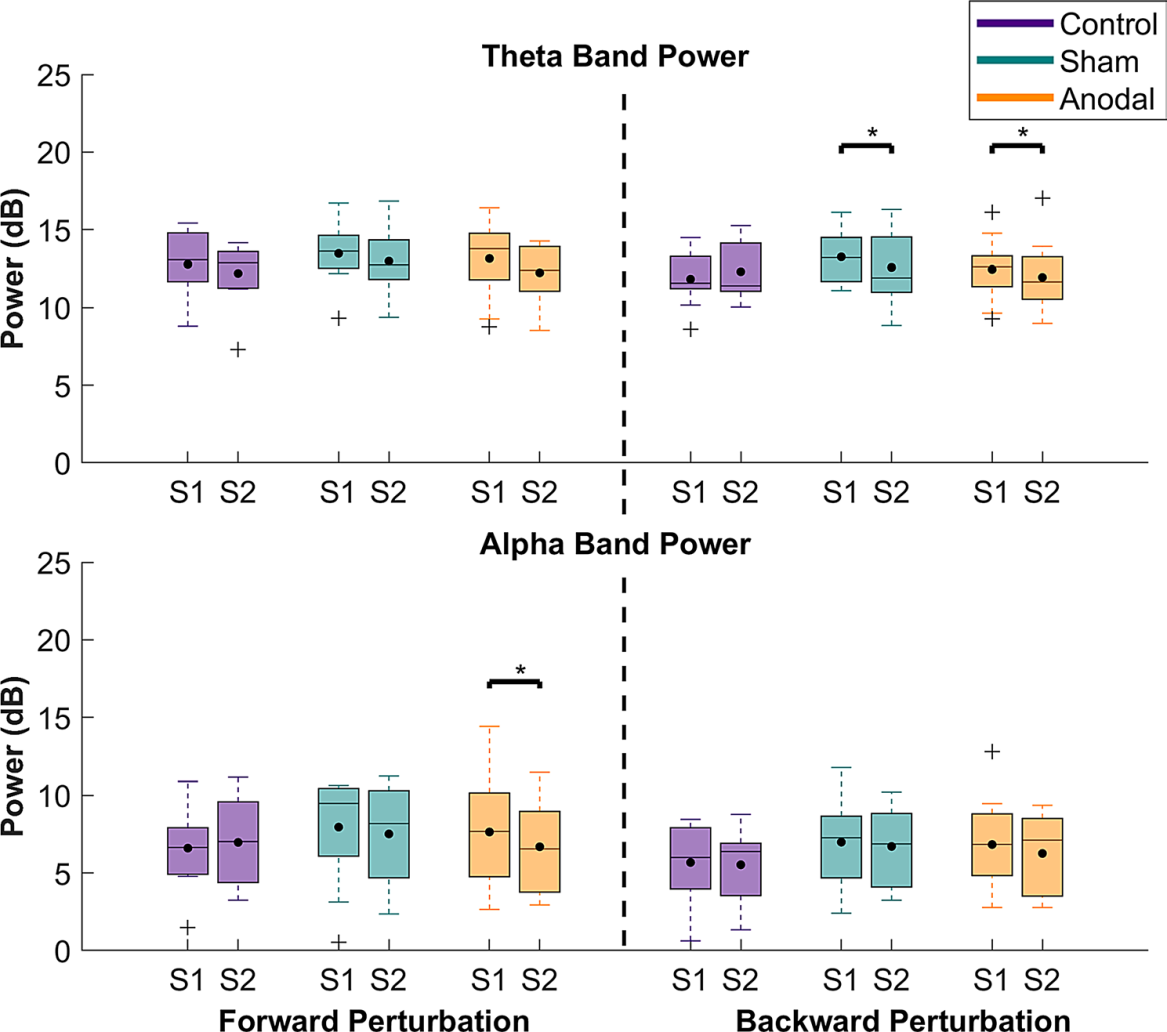
Average theta and alpha band power for each group and perturbation direction (averaged over the sound factor). Each band power was calculated using data from 90 to 250 ms after perturbation onset, relative to baseline (from 1000 to 200 ms before the perturbation onset). The time range of interest was determined according to previous studies showing that cortical involvement starts at ~ 90 ms, and stepping responses start at around 250 ms after perturbation onset [2, 10, 76]. S1 = Pre-training, S2 = Post-training

**Fig. 9:**
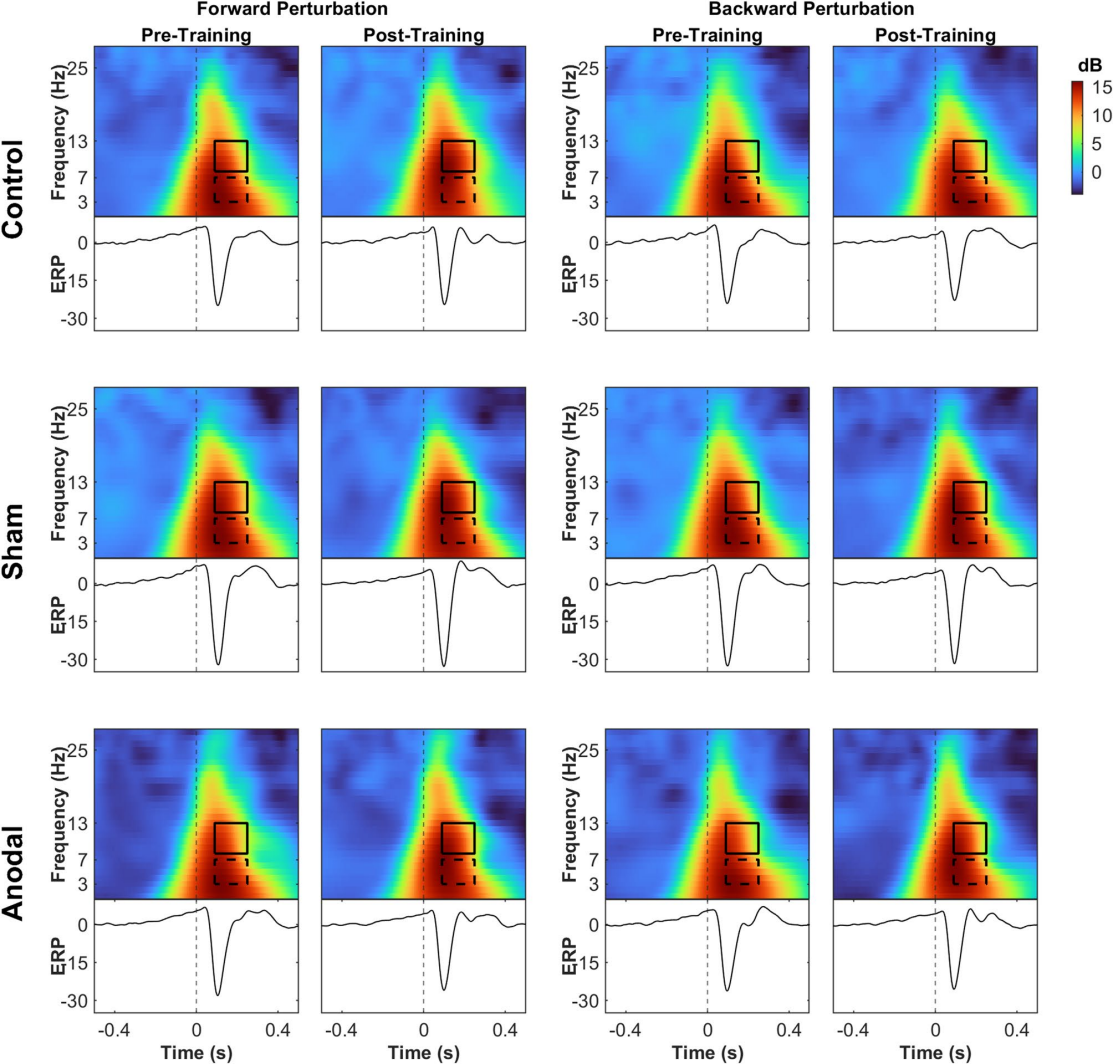
Characteristics of cortical responses to balance perturbations are depicted in the time-frequency and ERP components for the no-sound conditions. The vertical dashed line marks the onset of the perturbation. The time-frequency domain maps illustrate the average power modulations for each group and session, highlighting typical broadband power increases (represented by warm colors). The dashed boxes delineate the theta frequency range (3–7 Hz), and the solid boxes represent the alpha frequency range (8–13 Hz) in the temporal window of interest (90–250 ms after perturbation onset)

**Fig. 10:**
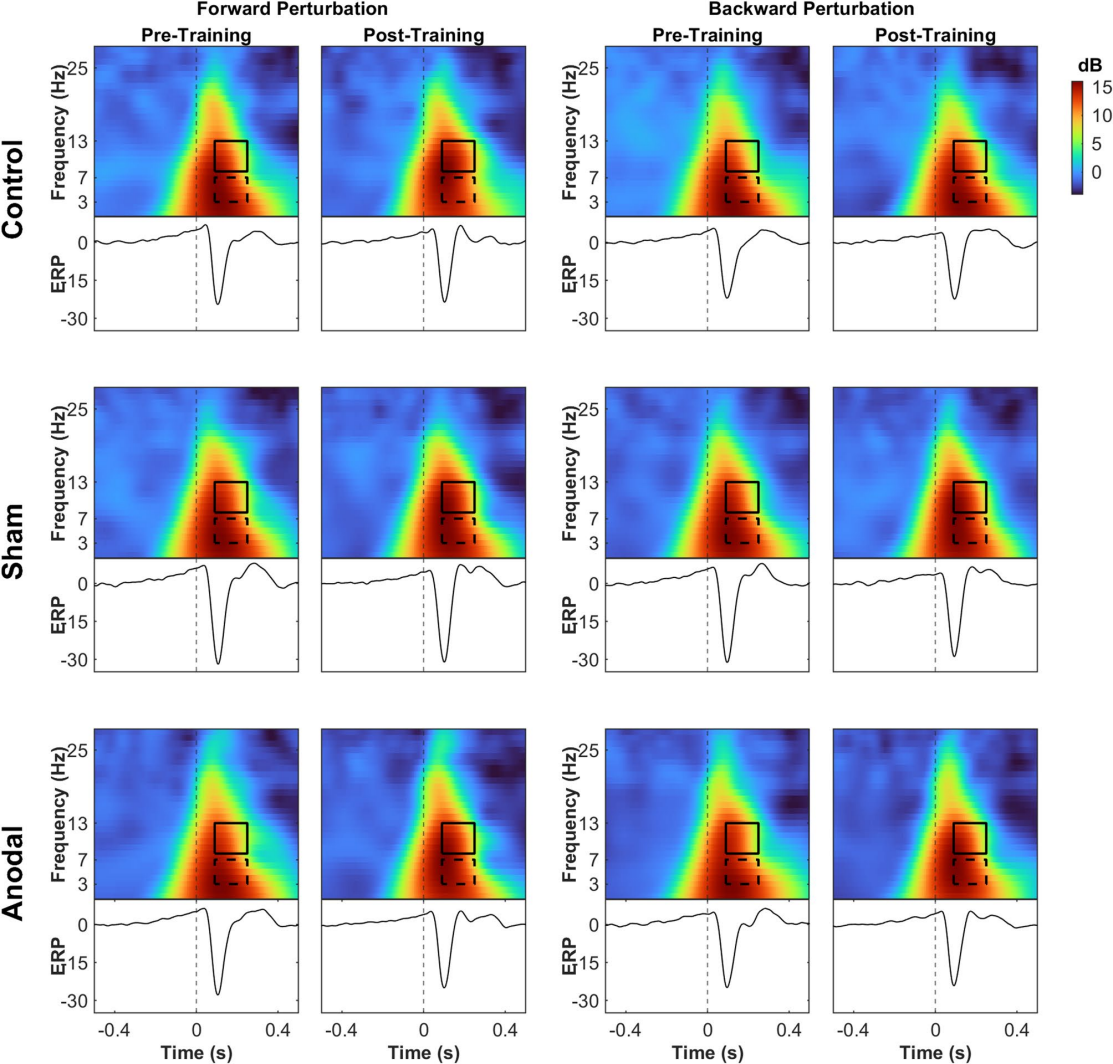
Characteristics of cortical responses to balance perturbations are depicted in the time-frequency and ERP components for the sound conditions. The vertical dashed line marks the onset of the perturbation. The time-frequency domain maps illustrate the average power modulations for each group and session, highlighting typical broadband power increases (represented by warm colors). The dashed boxes delineate the theta frequency range (3–7 Hz), and the solid boxes represent the alpha frequency range (8–13 Hz) in the temporal window of interest (90–250 ms after perturbation onset)

For the time x group interaction in the backward perturbation, training significantly reduced theta power in both sham and anodal stimulation groups as illustrated in Fig. 8 (*p* = 0.0132, d = 0.76, and *p* = 0.0485, d = 0.58 respectively, supplementary material Table 31). Alpha power decreased significantly in the anodal stimulation group after training, as shown in Fig. 8 (*p* = 0.007, d = 0.8, supplementary material Table 33). Figures 9 and 10 illustrate the EEG ERSPs and the N1 mean amplitude for each session and direction, for the no-sound and sound conditions, respectively.

### SICI/ICF

There was a significant main effect of muscle in both SICI and ICF (*p* = 0.00650, η_p_^2^ = 0.05, *p* = 0.00491, η_p_^2^ = 0.06, respectively), with small and medium effect sizes, respectively. A significant time x muscle interaction was revealed (*p* = 0.0486, η_p_^2^ = 0.04), with a small effect size. No additional main or interaction effect were observed (*p* = 0.222–0.858). Detailed statistics of the mixed-model ANOVA are shown in supplementary material, Table 5.

**Fig. 11:**
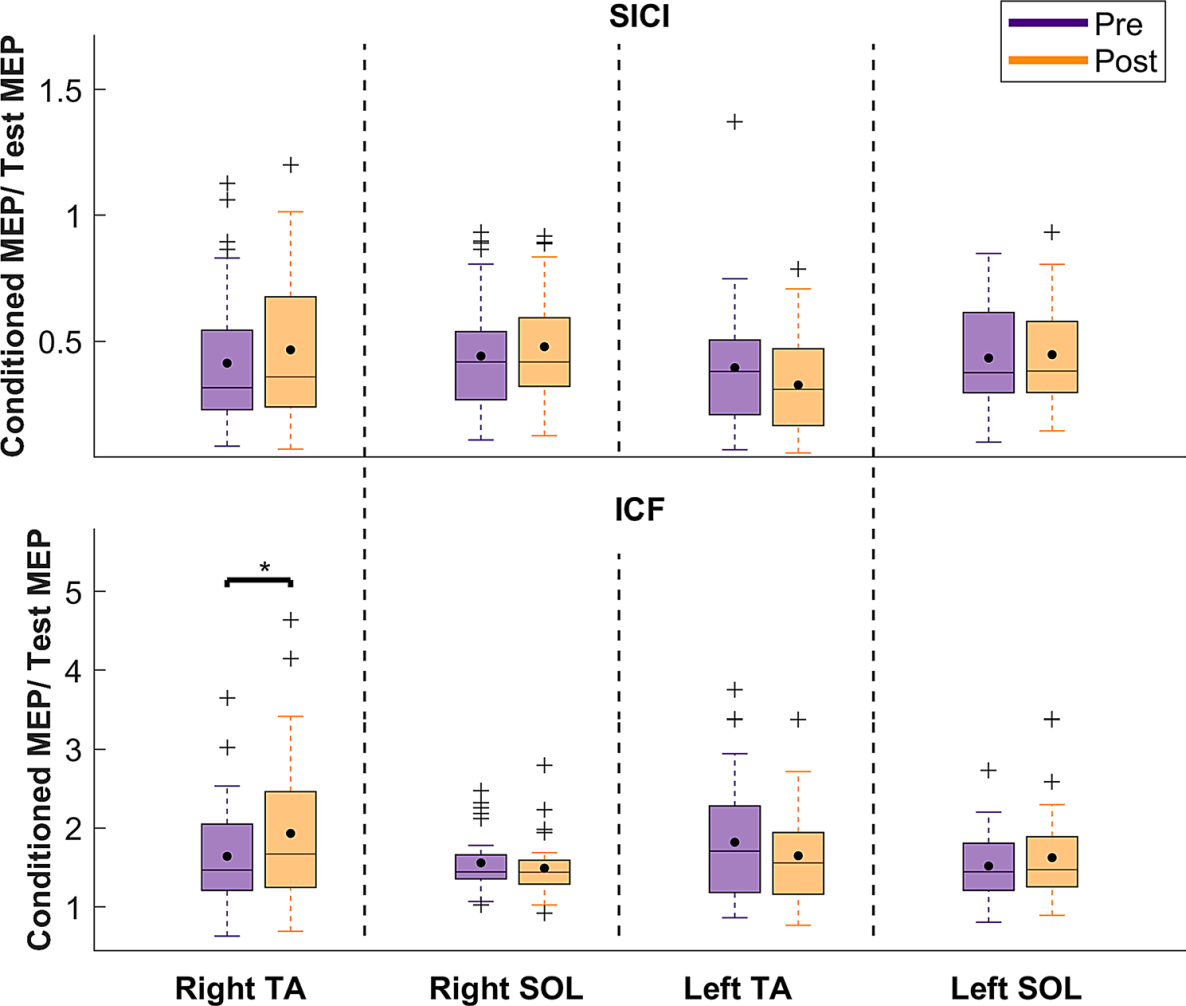
SICI and ICF were calculated using the formula conditioned MEP/Test MEP. Thus, smaller SICI values indicate stronger intracortical inhibition, whereas a higher ICF value reflects enhanced intracortical facilitation. Each box shows the interquartile range (25th-75th percentiles), and the horizontal line in each box indicates the median, while the black dot indicates the mean. Outliers are indicated with a “+” and whiskers represent the range of data without outliers. Asterisk indicates significant differences before and after training

For the main effect of muscle, post-hoc analyses revealed that the left TA showed a significantly higher inhibition than the right and left SOL (*p* = 0.0064, d = 0.56 and *p* = 0.0462, d = 0.45, respectively, supplementary material Table 36). The right TA showed a significantly higher facilitation compared to the right soleus (*p* = 0.0119, d = 0.14, supplementary material Table 37). For the time x muscle interaction, no statistically significant difference was found between the muscles in the pre-training session. However, following training, the right TA showed a higher ICF compared to the right SOL (*p* = 0.0019, d = 0.88, supplementary material Table 38). Besides, as illustrated in Fig. 11, ICF in the right TA muscle significantly increased after the perturbation-based balance training across all groups (*p* = 0.0196, d = 0.57, supplementary material Table 39).

## Discussion

This study examined changes in kinematic and cortical responses to balance perturbations and changes in corticospinal excitability after short-term balance training, including the effects of non-invasive brain stimulation with tDCS, to reveal the involvement of the primary motor cortex in early balance learning. Our findings indicate that combining balance training with anodal tDCS significantly improved balance performance in forward perturbations. Specifically, COM displacement and hip joint excursions on both sides were significantly reduced in the anodal stimulation group post-training, suggesting enhanced postural control mechanisms facilitated by the neuromodulatory effects of tDCS.

The CNS monitors COM movements, detects when these exceed desired boundaries, and initiates appropriate responses to regain balance, particularly during unexpected perturbations [81]. When balance is disturbed, proprioceptive signals arise from ankle trigger reflex responses originating from spinal and supraspinal structures (5). Rapid and robust responses and intermuscular coordination prevent the body from falling [4]. Thus, decreased COM displacement in forward perturbation indicates an enhanced ability to recover balance in response to perturbations, reflecting improved postural stability and balance control mechanisms in the anodal stimulation group. Supporting this, previous studies reported a reduction in peak COM displacement following perturbation-based balance training of varying durations, similar to the current study [15, 82].

We calculated the peak joint excursions after perturbation onset for the hip, knee, and ankle joints on both sides. The immediate reaction of the ankle joint compensates for the balance disturbance and promotes faster balance recovery [3, 83, 84]. When a perturbation strongly disturbs balance, the hip strategy, involving hip joint excursion to move the COM rapidly, is used to regain balance [10, 85]. The decrease in the right hip, knee, and ankle joint excursions observed after training, regardless of the intervention group, suggests that perturbation-based balance training improved balance control in response to forward perturbations across all participants. Furthermore, the reduced right and left hip excursions only in the anodal stimulation group in forward perturbations and the significant interaction effect imply that anodal tDCS combined with perturbation-based balance training further facilitated balance control. Moreover, the control group showed significantly larger joint excursions after training, and group comparisons showed a significant difference between the control and anodal stimulation group following training, suggesting higher stability and possibly faster responses to the perturbation in the left ankle in the anodal stimulation group. The predominant postural strategy following perturbation can be assessed by examining hip joint involvement [85]. Increased hip movement reflects reliance on a hip strategy due to greater perturbation impact, while reduced hip movement indicates sufficient balance recovery through the ankle strategy. Thus, after training, the anodal stimulation group likely shifted their balance recovery strategy from hip to ankle.

The peak joint excursions were expected to differ depending on the direction of the perturbation. For example, hip joints have a greater flexion range of motion than extension. Thus, forward perturbations may elicit larger joint excursions than backward perturbations. Similarly, the knee joint has a minimal joint extension while standing, but knee flexion can be used to recover balance rapidly by means of lowering the COM height as a balance recovery strategy [86]. Although we did not statistically compare the different perturbation directions, it was observed that both the hip and ankle joints had larger excursions during forward perturbations than during backward perturbations (Fig. 6). Furthermore, right knee joint excursions were significantly decreased across all participants in forward perturbations, suggesting enhanced balance ability following training. In contrast to the forward perturbations, both knee joint excursions increased in backward perturbations following training across all participants. These findings support the idea that different balance control strategies are used for different perturbation directions [13]. Balance training reduced the knee joint excursions in forward perturbations, suggesting increased stability for recovering balance. Conversely, it led to an increased knee joint excursion in backward perturbations, suggesting a more dynamic and flexible balance control strategy. Thus, behavioral adaptation to the training depends on the direction of the perturbation, highlighting how biomechanical characteristics of the body contribute to the need for different balance control strategies depending on the perturbation direction.

The body is in an equilibrium when the vertical projection of the COM on the ground falls within the base of support, the feet, while standing. When balance is disturbed, the COM shifts towards the boundary of the feet. The limit of stability is greater when leaning forward than backward, as the feet extend further in front than the back of the body [10]. This anatomical arrangement allows the plantar flexors, involving the soleus muscle, to generate a greater balance-recovering torque after backward perturbations. As a result, the body can more easily maintain the COM away from the neutral position during backward perturbations than during forward perturbations. Maintaining COM more stable might be a good strategy for backward perturbations. In contrast, a stiffer response, decreasing the COM displacement to avoid falling, might be more effective for forward perturbations because of the biomechanical properties of the body described above. In this context, the increased ICF in the right TA observed in the current study may reflect an enhanced neural drive to the TA muscle, facilitating faster compensatory responses contributing to reduced ankle joint excursion and decreased peak COM displacement after forward perturbations.

A previous study showed that forward perturbation and startle reflex may share a common mechanism to generate more rapid responses to perturbation [13]. These findings suggest that different neural circuits are likely involved in the sensorimotor processing of perturbations in different directions. The current study found no significant main or interaction effect for the N1 amplitude in forward perturbation. However, training significantly reduced the N1 amplitude in backward perturbation conditions across all participants. Supporting this finding, previous studies showed that individuals with impaired balance ability have a larger N1 response [20], which decreased through ten perturbation trials [22]. While training led to improved balance control, indicated by significantly reduced joint excursions in forward perturbations, there was no significant change in N1 amplitude. This suggests that, beyond improved balance functions, cognitive processes, such as perceived threat, might influence the N1 amplitude in conjunction with balance ability.

Indeed, the N1 amplitude is affected by cognitive components, including attention [87], perceived threat [88, 89], and predictability of postural perturbations [88–92]. Previous studies defined its role in sensory integration, error detection, and movement initiation [16, 28], even though the exact mechanisms remain unclear. Cognitive components of the N1 response suggest that the balance recovery process becomes more automatic through training, and perturbations are no longer perceived as significant postural threats, diminishing the need for enhanced attention. These findings are consistent with previous studies indicating a transition of balance control from cortical to subcortical structures during balance training, leading to more automated responses to perturbations. In young adults, balance training has been shown to reduce spinal excitability [93]. Although we did not explicitly assess spinal excitability in the present study, the improved balance recovery observed here may be primarily mediated by subcortical or cerebellar structures interacting with M1.

Our findings regarding the N1 amplitude indicate that forward perturbations generate a more prominent postural threat even after training, possibly because forward perturbations are more challenging than backward perturbations. Therefore, the participants may commit more cognitive resources to sensory integration or attention.

In line with the current findings, a previous study demonstrated that decreased N1 latency occurred when perturbation intensity and probability of a stepping response increased, regardless of the perturbation direction. The authors suggested that increasing computational load before determining the need for a stepping response is associated with decreased N1 latency, since reduced latency enables a longer time to generate longer latency responses such as stepping [8]. If more challenging perturbations lead to decreased N1 latency, it would be expected that balance training would not reduce latency since the perturbations are likely to be more easily handled after training. However, EEG responses were assessed over Cz in the current study, suggesting that the observed N1 responses predominantly reflect activity in M1 rather than in the primary somatosensory cortex (S1) [94]. Therefore, reduced N1 latency in both perturbation directions after training across all participants suggests faster motor processing. Notably, only the anodal stimulation group showed further reduction in N1 latency in backward perturbations, which indicates a modulatory effect of tDCS on N1 latency. This finding may indicate improved efficiency of the balance responses to backward perturbations, specifically in the anodal stimulation group, even though no significant behavioral improvement was observed.

Furthermore, we observed significant modulations in theta and alpha band power in the time bin 90 and 250 ms after perturbation onset. Specifically, theta band power decreased in both sham and anodal stimulation groups during backward perturbations and across all groups in forward perturbations, implying that balance training, regardless of the type of stimulation, influenced cortical processes, possibly related to enhanced sensory integration and motor output. However, alpha band power was reduced in forward perturbations only in the anodal stimulation group after training, potentially reflecting enhanced sensory processing in the forward direction facilitated by tDCS. These findings suggest that anodal tDCS over M1 may modulate sensorimotor processing, facilitating balance ability.

Sensorimotor processing is associated with the N1 amplitude and underlying modulations of theta and alpha rhythms [18, 26, 95]. Supporting this, a previous study suggested that theta band power scales with perturbation intensity [27] and its impact on balance. When the body leans forward before a backward perturbation, amplifying the impact of the perturbation, theta power is increased more quickly [27]. This increase in theta power may represent error detection between the current and desired posture and may precede the level of long latency response behaviors after perturbation [8, 27]. It was demonstrated that theta power increases in the instance of balance loss, supporting its role in balance monitoring. In addition, Peterson and Ferris found increased theta power connectivity between SMA and several other brain regions involving bilateral sensorimotor and anterior cingulate cortices, which refers to the interaction between various brain regions during a standing balance task. Their finding indicates a possible contribution of various brain regions to the N1 response and increased sensorimotor processing during balance recovery [96]. The SMA serves as a hub for theta band connectivity with other motor areas [96] and for sensory integration with connections to the prefrontal cortex [95] and brainstem [97].

The results of the present study support these previous findings, suggesting theta rhythms may be involved in the error detection process and cognitive control during balance disturbances [8], as decreased theta power was observed in both directions following training without a specific effect of tDCS over M1. Alpha power, on the other hand, is known for its involvement in sensorimotor processing [8, 19] and is also associated with balance control, possibly reflecting motor preparation and execution of reactive responses to balance disturbances [8, 19, 28]. Therefore, the observed decrease in alpha power after training only in the anodal stimulation group implies an improved motor adaptation, possibly through strengthening communication between the SMA and motor cortices, resulting in better balance during forward perturbations.

While decreased inhibition and enhanced cortical excitability are observed after motor training, balance training induced partially opposite changes. Previous studies have shown a reduction in cortical contributions during balance training, indicated by decreased corticospinal and cortical excitability and increased SICI [30]. A few studies reported intracortical excitability changes after balance training [39, 40, 42, 43, 98]. Mouthon & Taube found an enhanced level of SICI during a balance test after two weeks of balance training [39]. This effect was confirmed by a study that compared the effect of 4 weeks of different types of training on cortical adaptation. They found increased SICI after balance training and reduced SICI after explosive training [40]. The increased SICI of the soleus muscle after four weeks of balance training returned to baseline levels following an additional four weeks of ballistic strength training [98]. SICI was measured during backward surface translation in all sessions. It is important to note that the findings mentioned above suggest that these cortical adaptations were observed when tested during task execution or while standing [39, 40, 42, 98], not at rest or during non-practiced tasks, which suggests these changes depend on the testing conditions. Similar SICI enhancements after long-term conventional balance training were observed in older people’s right TA muscle [99, 100]. In contrast to SICI measurements, only two studies (with one and sixteen balance training sessions) examined ICF and found no significant change at rest or during standing [42, 43]. In the current study, however, training induced increased ICF in the right TA muscle across all participants. Although previous results showed increased inhibition and decreased facilitation after balance training tested after at least 2 weeks of training, increased excitability might be required in the initial phase of the training to support long-term potentiation (LTP). LTP is largely dependent on N-methyl-D-aspartate (NMDA) receptors, which are calcium (Ca^2+^) channels activated by glutamate [101]. The change in ICF potentially indicates an enhancement in glutamatergic neurotransmission in M1.

It should also be noted that the ICF patterns differ between the right and left TA muscles. Specifically, after training, ICF was significantly increased in the right TA muscle, while the left TA showed a trendwise decreased ICF (*p* = 0.177, supplementary material Table 39). Moreover, significant improvements in joint excursions after training were only observed on the right side (hip, knee, and ankle), indicating distinct behavioral adaptations on both sides. This observation may imply that bilateral balance training may stimulate adaptation differently in both hemispheres.

Furthermore, the above-mentioned studies investigating intracortical excitability in balance skill acquisition conducted conventional but not perturbation-based balance training, which is more challenging and requires faster sensory processing and motor output [39, 40, 43, 98]. Therefore, the findings of the present study may differ from those of previous ones because of the difference in the type of balance training.

This study shows that transcranial Direct Current Stimulation (tDCS) targeting the motor cortex (M1) enhances cortical and behavioral outcome measures in young adults, particularly in response to forward perturbations that lead to posterior balance disturbances. Improvements in balance performance and behavior-related physiological changes were observed across all groups following training, as indicated by a significant main effect of time. These included decreased right knee and ankle joint excursions in forward perturbations and increased right and left knee joint excursions in backward perturbations. Additionally, reductions in average theta power and N1 latency were observed in forward perturbation, along with a decrease in N1 amplitude in backward perturbation. Furthermore, an increase in ICF in the right tibialis anterior muscle was observed after training.

In addition to the effects of training alone, tDCS applied over M1 concurrently with balance training significantly improved short-term balance skill acquisition. This was reflected in greater improvements in balance performance, observed only in the anodal stimulation group, including decreased bilateral hip joint excursions and reduced center of mass (COM) displacement, as well as decreased alpha power, and shorter N1 latency in response to forward perturbations post-training compared to pre-training.

A longer training period or larger sample size may be necessary to detect statistically significant pre- to post-training changes in all groups or to reveal significant between-group differences post intervention. This is consistent with previous studies that implemented longer periods of perturbation-based balance training [3, 15, 102, 103] and with a recent review highlighting that the effects of training duration remain unclear [104]. These findings suggest that anodal stimulation over M1 may accelerate the balance learning process, highlighting the role of M1 in short-term balance training.

Furthermore, the acoustic signal, given two seconds before the perturbation, had heterogeneous effects on COM displacement depending on perturbation direction. Specifically, acoustic signals decreased COM displacement in forward perturbations while increasing it in backward perturbations. This directional difference in response to the acoustic signal may be attributed to the postural control strategy. When individuals receive a warning signal before perturbation, they may intentionally lean forward, anticipating a forward perturbation. These anticipatory adjustments are likely since backward perturbations can be easily handled due to the biomechanical factors mentioned earlier. Although the acoustic signal provided a timing cue for the onset of the perturbation, it did not convey any directional information to the participants. This may have influenced the effect of the acoustic signal based on the perturbation direction, possibly due to distinct neural processing of different perturbation directions [13]. In the current study, preparing for the perturbation after the acoustic signal caused more flexible reactions with increased COM displacements and accompanying decreased N1 amplitude and underlying frequency band power in backward perturbations. In the forward perturbations, on the other hand, the acoustic signal caused a decrease in only theta band power, possibly because of a decreased error between expected and current body posture with preparation for the perturbation. The missing interaction between sound and other factors indicates that cortical responses to the anticipated perturbations did not significantly change through perturbation-based balance training or tDCS intervention.

## Conclusion

Our findings suggest that balance training induces significant changes in cortical and behavioral parameters regardless of the intervention groups. Specifically, we found decreased hip, knee, and ankle joint excursions in forward perturbations as well as increased knee joint excursions in backward perturbations, demonstrating improved balance control across all participants. These improvements were supported by cortical adaptations measured by decreased N1 amplitude, latency, and average theta power. We also found a significantly enhanced ICF in the right TA muscle. Only anodal tDCS showed significant improvement of COM displacement and hip joint excursions in the forward direction. Supporting these behavioral findings, we also observed a reduction in alpha band power during the forward perturbations following training combined with anodal stimulation. These results suggest that anodal tDCS partially enhances the effects of perturbation-based balance training by modulating sensorimotor processing and facilitating neuroplasticity in M1.

### Limitations

Potential limitations of this study should be taken into account. First, we used the same perturbation displacement, meaning the same acceleration and speed of perturbation stimulus, in both directions to elicit a similar proprioceptive signal. However, this may mean that backward perturbations are not sufficiently challenging, possibly causing a ceiling effect in the behavioral analysis. Second, these findings may not directly be transferred to older adults or people with balance-affecting disorders. Third, we did not measure SICI and ICF during task performance, which limits our ability to observe the full range of inhibition and facilitation between active and passive conditions. Assessing cortical excitability only at rest may lead to misinterpretations regarding the cortical adaptations that occur during balance tasks. Fourth, the limited timeline and the necessity to exclude some participants (e.g., those not meeting MEP amplitude criteria) led to a smaller sample size than expected. However, the data were analyzed with a linear mixed-effects model, which accounts for inter-subject variability. Finally, we did not assess blinding or side effects following tDCS applications, and thus could not rule out the presence of placebo effects.

### Future directions

The findings of this study support previous reports that suggest a central role of M1 in balance skill acquisition. However, the acquisition and retention of balance skills may also depend on other cortical or subcortical regions that communicate with M1, such as the basal ganglia, cerebellum, or SMA. In this context, brain modulation techniques remain essential to reveal the contribution of these structures to balance learning. Future studies should explore the effects of combined stimulation of M1 and other brain regions to elucidate the underlying neural mechanisms of reactive balance control. Developing combined stimulation protocols that induce strong and prolonged clinically relevant effects is important for future approaches. In addition to that, balance learning with tDCS over M1 in other populations, such as people with Parkinson’s disease, would enhance knowledge about the role of M1 in balance learning and provide critical information about the transferability of results.

## Electronic supplementary material

Below is the link to the electronic supplementary material.

Supplementary Material 1

## Data Availability

On reasonable request, the datasets generated during the present study are available from the corresponding author.

## Abbreviations

M1: Primary Motor Cortex
tDCS: Transcranial Direct Current Stimulation
EEG: Electroencephalography
COM: Center of Mass
SICI: Short-Interval Intracortical Inhibition
TMS: Transcranial Magnetic Stimulation
ICF: Intracortical Facilitation
TA: Tibialis Anterior
SOL: Soleus
CNS: Central Nervous System
MEP: Motor-Evoked Potential
EMG: Electromyography
PAR-Q: Physical Activity Readiness Questionnaire
rMT: Resting Motor Threshold
GRAIL: Gait Real-Time Analysis Interactive Laboratory
HBM: Human Body Model
ICA: Independent Component Analysis
ERSP: Event-Related Spectral Perturbation
ERP: Event-Related Potential
S1: Primary Somatosensory Cortex
LTP: Long-Term Potentiation
NMDA: N-methyl-D-aspartate
