# Supplementary material for: Effect of transcranial direct current stimulation over the primary motor cortex on short-term balance acquisition in healthy individuals
Source: J Neuroeng Rehabil. 2025 Jul 4;22:146. doi: 10.1186/s12984-025-01663-3 (PMC12232060; doi:10.1186/s12984-025-01663-3)
Supplement: Supplementary file 1 — Supplementary Material 1 [file 12984_2025_1663_MOESM1_ESM.docx]

**Table 1** Mixed-ANOVA results for the effect of tDCS on COM displacements for each perturbation direction.

|  | Main/Interaction | **df** | **F value** | **p** | **η_p_^2^** |
| --- | --- | --- | --- | --- | --- |
| Forward Perturbation | Time | 1 | 0.68 | 0.411 | 0.01 |
|  | Sound | 1 | 8.61 | 0.00422* | 0.09 |
|  | Group | 2 | 1.11 | 0.343 | 0.07 |
|  | Time x Sound | 1 | 1.21 | 0.274 | 0.01 |
|  | Time x Group | 2 | 3.10 | 0.0497* | 0.06 |
|  | Sound x Group | 2 | 0.15 | 0.861 | 0.00 |
|  | Time x Sound x Group | 2 | 0.85 | 0.432 | 0.02 |
| Backward Perturbation | Time | 1 | 3.67 | 0.0586 | 0.01 |
|  | Sound | 1 | 12.90 | 0.00053* | 0.09 |
|  | Group | 2 | 1.60 | 0.217 | 0.07 |
|  | Time x Sound | 1 | 0.07 | 0.794 | 0.01 |
|  | Time x Group | 2 | 0.51 | 0.600 | 0.06 |
|  | Sound x Group | 2 | 3.06 | 0.0517 | 0.00 |
|  | Time x Sound x Group | 2 | 0.41 | 0.663 | 0.02 |

df: degrees of freedom, η_p_^2^: partial eta squared, F: F-value. Significant results are indicated by asterisks *.

**Table 2.** Mixed-ANOVA results for the effect of tDCS on the peak excursions of lower extremity joints.

|  |  | **Right Hip** | | | **Left Hip** | | | **Right Knee** | | | **Left Knee** | | | **Right Ankle** | | | **Left Ankle** | | |
| --- | --- | --- | --- | --- | --- | --- | --- | --- | --- | --- | --- | --- | --- | --- | --- | --- | --- | --- | --- |
| **Forward Perturbation** | **df** | **F** | **p** | **η_p_^2^** | **F** | **p** | **η_p_^2^** | **F** | **p** | **η_p_^2^** | **F** | **p** | **η_p_^2^** | **F** | **p** | **η_p_^2^** | **F** | **p** | **η_p_^2^** |
| Time | 1 | 6.06 | 0.0160* | 0.07 | 0.49 | 0.487 | 0.01 | 12.95 | 0.000555* | 0.14 | 0.40 | 0.531 | 0.005 | 5.40 | 0.0227* | 0.06 | 0.29 | 0.590 | 0.004 |
| Sound | 1 | 0.04 | 0.555 | 0.00 | 0.02 | 0.518 | 0.01 | 0.02 | 0.547 | 0.005 | 0.00 | 0.370 | 0.01 | 0.13 | 0.810 | 0.001 | 0.08 | 0.731 | 0.001 |
| Group | 2 | 1.81 | 0.547 | 0.04 | 1.03 | 0.359 | 0.07 | 1.54 | 0.700 | 0.03 | 1.49 | 0.476 | 0.05 | 1.38 | 0.679 | 0.03 | 2.88 | 0.0890 | 0.16 |
| Time x Sound | 1 | 0.00 | 0.993 | 0.00 | 0.00 | 0.948 | 0.0001 | 0.00 | 0.958 | 0.00 | 0.06 | 0.809 | 0.001 | 0.09 | 0.765 | 0.001 | 0.17 | 0.680 | 0.002 |
| Time x Group | 2 | 4.98 | 0.00916* | 0.11 | 4.30 | 0.0168* | 0.10 | 2.25 | 0.112 | 0.05 | 2.55 | 0.0847 | 0.06 | 2.83 | 0.0652 | 0.07 | 7.04 | 0.00154* | 0.15 |
| Sound x Group | 2 | 0.19 | 0.821 | 0.00 | 0.28 | 0.752 | 0.01 | 0.29 | 0.832 | 0.005 | 0.24 | 0.766 | 0.01 | 0.09 | 0.889 | 0.00 | 0.02 | 0.997 | 0.0001 |
| Time x Sound x Group | 2 | 0.21 | 0.811 | 0.01 | 0.17 | 0.840 | 0.004 | 0.19 | 0.825 | 0.005 | 0.16 | 0.850 | 0.004 | 0.04 | 0.961 | 0.00 | 0.02 | 0.983 | 0.0004 |
| **Backward Perturbation** |  |  | | |  | | |  | | |  | | |  | | |  | | |
| Time | 1 | 2.07 | 0.154 | 0.03 | 3.25 | 0.0752 | 0.04 | 13.41 | 0.000451* | 0.14 | 17.15 | <0.0001* | 0.18 | 3.19 | 0.0778 | 0.04 | 1.14 | 0.289 | 0.01 |
| Sound | 1 | 1.00 | 0.320 | 0.01 | 0.61 | 0.438 | 0.01 | 0.08 | 0.777 | 0.001 | 0.30 | 0.584 | 0.004 | 0.05 | 0.829 | 0.001 | 0.01 | 0.925 | 0.0001 |
| Group | 2 | 0.05 | 0.951 | 0.004 | 0.42 | 0.662 | 0.03 | 1.29 | 0.293 | 0.09 | 0.87 | 0.430 | 0.06 | 0.35 | 0.710 | 0.02 | 0.42 | 0.664 | 0.03 |
| Time x Sound | 1 | 0.03 | 0.861 | 0.0004 | 0.85 | 0.358 | 0.01 | 0.03 | 0.872 | 0.0003 | 0.05 | 0.825 | 0.001 | 0.61 | 0.437 | 0.01 | 0.44 | 0.511 | 0.01 |
| Time x Group | 2 | 0.52 | 0.597 | 0.01 | 2.41 | 0.0964 | 0.06 | 0.60 | 0.551 | 0.01 | 0.55 | 0.577 | 0.01 | 1.80 | 0.172 | 0.04 | 0.45 | 0.639 | 0.01 |
| Sound x Group | 2 | 0.05 | 0.952 | 0.001 | 0.07 | 0.934 | 0.002 | 0.02 | 0.979 | 0.001 | 0.02 | 0.983 | 0.0004 | 0.16 | 0.856 | 0.004 | 0.04 | 0.957 | 0.001 |
| Time x Sound x Group | 2 | 0.19 | 0.830 | 0.005 | 0.17 | 0.840 | 0.004 | 0.11 | 0.897 | 0.003 | 0.66 | 0.517 | 0.02 | 0.06 | 0.941 | 0.002 | 0.99 | 0.378 | 0.02 |

df: degrees of freedom, η_p_^2^: partial eta squared, F: F-value. Significant results are indicated by asterisks *.

**Table 3.** Mixed-ANOVA results for the effect of tDCS on N1 responses

|  |  |  |  | **Cz Amplitude** | | | **Cz Latency** | | |
| --- | --- | --- | --- | --- | --- | --- | --- | --- | --- |
|  |  | Main/Interaction | **df** | **F** | **p** | **η_p_^2^** | **F** | **p** | **η_p_^2^** |
| Forward Perturbation |  | Time | 1 | 3.56 | 0.0625 | 0.04 | 37.60 | <0.0001* | 0.29 |
|  |  | Sound | 1 | 1.05 | 0.308 | 0.01 | 0.00 | 0.984 | 0.00 |
|  |  | Group | 1 | 2.47 | 0.101 | 0.14 | 0.24 | 0.792 | 0.02 |
|  |  | Time x Sound | 2 | 0.51 | 0.476 | 0.01 | 0.66 | 0.419 | 0.01 |
|  |  | Time x Group | 1 | 1.42 | 0.248 | 0.03 | 0.91 | 0.406 | 0.02 |
|  |  | Sound x Group | 1 | 0.07 | 0.934 | 0.002 | 0.77 | 0.468 | 0.02 |
|  |  | Time x Sound x Group | 1 | 0.02 | 0.984 | 0.0004 | 0.18 | 0.839 | 0.004 |
| Backward Perturbation |  | Time | 2 | 8.85 | 0.00376* | 0.09 | 17.09 | <0.0001* | 0.16 |
|  |  | Sound | 2 | 11.53 | 0.00102* | 0.11 | 0.52 | 0.473 | 0.01 |
|  |  | Group | 2 | 3.54 | 0.0417* | 0.19 | 0.22 | 0.808 | 0.01 |
|  |  | Time x Sound | 1 | 0.01 | 0.910 | 0.007 | 1.08 | 0.302 | 0.01 |
|  |  | Time x Group | 2 | 0.33 | 0.721 | 0.01 | 3.62 | 0.0308* | 0.07 |
|  |  | Sound x Group | 2 | 0.91 | 0.408 | 0.02 | 1.19 | 0.310 | 0.03 |
|  |  | Time x Sound x Group | 2 | 0.60 | 0.550 | 0.01 | 0.20 | 0.820 | 0.004 |

df: degrees of freedom, η_p_^2^: partial eta squared, F: F-value. Significant results are indicated by asterisks *.

**Table 4.** Mixed-ANOVA results for the effect of tDCS on theta and alpha band power.

|  | | | **Averaged Theta Power**  **(3-7 Hz)** | | | **Averaged Alpha Power**  **(8-13 Hz)** | | |
| --- | --- | --- | --- | --- | --- | --- | --- | --- |
|  | Main/Interaction | **df** | **F** | **p** | **η_p_^2^** | **F** | **p** | **η_p_^2^** |
| Forward Perturbation | Time | 1 | 19.92 | <0.0001* | 0.18 | 2.65 | 0.107 | 0.03 |
|  | Sound | 1 | 3.96 | 0.0496* | 0.04 | 2.25 | 0.137 | 0.02 |
|  | Group | 1 | 0.40 | 0.672 | 0.03 | 0.27 | 0.767 | 0.02 |
|  | Time x Sound | 2 | 1.09 | 0.299 | 0.01 | 0.57 | 0.454 | 0.01 |
|  | Time x Group | 1 | 0.74 | 0.479 | 0.02 | 3.35 | 0.0395* | 0.07 |
|  | Sound x Group | 1 | 0.28 | 0.758 | 0.01 | 0.42 | 0.657 | 0.01 |
|  | Time x Sound x Group | 1 | 0.40 | 0.672 | 0.01 | 0.29 | 0.748 | 0.01 |
| Backward Perturbation | Time | 2 | 2.31 | 0.132 | 0.03 | 2.97 | 0.0885 | 0.03 |
|  | Sound | 2 | 8.98 | 0.00354* | 0.09 | 9.18 | 0.0032* | 0.09 |
|  | Group | 2 | 0.63 | 0.542 | 0.04 | 0.71 | 0.502 | 0.04 |
|  | Time x Sound | 1 | 0.88 | 0.352 | 0.01 | 0.31 | 0.578 | 0.003 |
|  | Time x Group | 2 | 5.20 | 0.007* | 0.10 | 0.44 | 0.647 | 0.01 |
|  | Sound x Group | 2 | 0.66 | 0.518 | 0.01 | 0.17 | 0.841 | 0.004 |
|  | Time x Sound x Group | 2 | 0.19 | 0.824 | 0.004 | 0.91 | 0.408 | 0.02 |

df: degrees of freedom, η_p_^2^: partial eta squared, F: F-value. Significant results are indicated by asterisks *.

**Table 5.** Mixed-ANOVA for the effect of tDCS on intracortical excitability.

|  |  | **df** | **F** | **p** | **η_p_^2^** |
| --- | --- | --- | --- | --- | --- |
| **SICI** | Time | 1 | 0.15 | 0.698 | 0.001 |
|  | Group | 2 | 1.00 | 0.379 | 0.06 |
|  | Muscle | 3 | 4.20 | 0.00650* | 0.05 |
|  | Time x Group | 2 | 0.15 | 0.858 | 0.001 |
|  | Time x Muscle | 3 | 1.48 | 0.222 | 0.02 |
|  | Group x Muscle | 6 | 1.25 | 0.284 | 0.03 |
|  | Time x Group x Muscle | 6 | 0.56 | 0.765 | 0.02 |
| **ICF** | Time | 1 | 0.42 | 0.518 | 0.002 |
|  | Group | 2 | 0.72 | 0.496 | 0.04 |
|  | Muscle | 3 | 4.41 | 0.00491* | 0.06 |
|  | Time x Group | 2 | 0.20 | 0.818 | 0.002 |
|  | Time x Muscle | 3 | 2.67 | 0.0486* | 0.04 |
|  | Group x Muscle | 6 | 1.28 | 0.266 | 0.03 |
|  | Time x Group x Muscle | 6 | 0.81 | 0.562 | 0.02 |

df: degrees of freedom, η_p_^2^: partial eta squared, F: F-value. Significant results are indicated by asterisks *.

**Post-Hoc Comparisons**

Post-hoc comparisons were illustrated for each outcome measure if a main or interaction effect was observed in the respective ANOVAs. Outcome measures were 1) Peak Center of Mass Displacement, 2) Joint Excursions, 3) N1 Amplitude, 4) N1 Latency, 5) Electroencephalography Frequency Bands Power and 6) Intracortical Excitability. Post-hoc comparisons of all outcome measures except intracortical excitability were made for both directions of balance perturbations separately, forward and backward perturbations.

# **PEAK CENTER OF MASS DISPLACEMENT**

**FORWARD PERTURBATION**

**Table 6** Post-hoc comparisons between sound conditions for all participants (Results are averaged over the levels of Time and Group). Each comparison has an Estimate (Estimated difference) standard error (SE), t-value, p-value, and Cohen’s d. Significant contrasts are indicated by asterisks *.

|  | **Estimate** | **SE** | **t** | **p** | **Cohen’s d** |
| --- | --- | --- | --- | --- | --- |
| No Sound - Sound | 0.88 | 0.30 | 2.93 | 0.0042* | 0.51 |

**Table 7.** Post-hoc comparisons between time points for each group (Results are averaged over the level of Sound). Each comparison has an Estimate (Estimated difference) standard error (SE), t-value, p-value, and Cohen’s d. Significant contrasts are indicated by asterisks *.

|  |  | **Estimate** | **SE** | **t** | **p** | **Cohen’s d** |
| --- | --- | --- | --- | --- | --- | --- |
| Pre-Post | Control | -0.62 | 0.52 | -1.19 | 0.237 | -0.36 |
|  | Sham | 0.17 | 0.52 | 0.33 | 0.742 | 0.10 |
|  | Anodal | 1.18 | 0.51 | 2.33 | 0.0219* | 0.69 |

**Table 8.** Post-hoc comparisons between groups in both time points (Results are averaged over the level of Sound). Each comparison has an Estimate (Estimated difference) standard error (SE), t-value, p-value, and Cohen’s d. Significant contrasts are indicated by asterisks *.

|  | **Pre** | | | |  | **Post** | | | |  |
| --- | --- | --- | --- | --- | --- | --- | --- | --- | --- | --- |
|  | **Estimate** | **SE** | **t** | **p** | **Cohen’s d** | **Estimate** | **SE** | **t** | **p** | **Cohen’s d** |
| Control –  Sham | -1.45 | 0.85 | -1.72 | 0.209 | -0.85 | -0.67 | 0.85 | -0.79 | 0.714 | -0.39 |
| Control –  Anodal | -1.09 | 0.83 | -1.30 | 0.40 | -0.63 | 0.72 | 0.83 | 0.86 | 0.669 | 0.42 |
| Sham –  Anodal | 0.37 | 0.83 | 0.44 | 0.898 | 0.21 | 1.38 | 0.83 | 1.66 | 0.233 | 0.80 |

**BACKWARD PERTURBATION**

**Table 9.**  Post-hoc comparisons between directions for all participants (Results are averaged over the levels of Time and Group). Each comparison has an Estimate (Estimated difference) standard error (SE), t-value, p-value, and Cohen’s d. Significant contrasts are indicated by asterisks *.

|  | **Estimate** | **SE** | **t** | **p** | **Cohen’s d** |
| --- | --- | --- | --- | --- | --- |
| No Sound - Sound | -1.04 | 0.29 | -3.6 | 0.0005* | -1.04 |

# **JOINT EXCURSIONS**

**FORWARD PERTURBATION**

## **Right Hip Joint**

**Table 10.** Post-hoc comparisons between time points for all participants (Results are averaged over the levels of Sound and Group). Each comparison has an Estimate (Estimated difference) standard error (SE), t-value, p-value, and Cohen’s d. Significant contrasts are indicated by asterisks *.

|  | **Estimate** | **SE** | **t** | **p** | **Cohen’s d** |
| --- | --- | --- | --- | --- | --- |
| Pre-Post | 0.91 | 0.37 | 2.46 | 0.016* | 0.45 |

**Table 11.** Post-hoc comparisons between time points for each group (Results are averaged over the level of Sound). Each comparison has an Estimate (Estimated difference) standard error (SE), t-value, p-value, and Cohen’s d. Significant contrasts are indicated by asterisks *.

|  |  | **Estimate** | **SE** | **t** | **p** | **Cohen’s d** |
| --- | --- | --- | --- | --- | --- | --- |
| Pre-Post | Control | -0.23 | 0.66 | -0.35 | 0.73 | -0.12 |
|  | Sham | 0.46 | 0.63 | 0.73 | 0.466 | 0.23 |
|  | Anodal | 2.49 | 0.62 | 4.04 | 0.0001* | 1.25 |

**Table 12.** Post-hoc comparisons between groups in both time points (Results are averaged over the level of Sound). Each comparison has an Estimate (Estimated difference) standard error (SE), t-value, p-value, and Cohen’s d. Significant contrasts are indicated by asterisks *.

|  | **Pre** | | | |  | **Post** | | | |  |
| --- | --- | --- | --- | --- | --- | --- | --- | --- | --- | --- |
|  | **Estimate** | **SE** | **t** | **p** | **Cohen’s d** | **Estimate** | **SE** | **t** | **p** | **Cohen’s d** |
| Control –  Sham | -2.51 | 2.12 | -1.18 | 0.471 | -1.26 | -1.82 | 2.12 | -0.86 | 0.67 | -0.91 |
| Control –  Anodal | -1.86 | 2.08 | -0.89 | 0.649 | -0.93 | 0.86 | 2.08 | 0.41 | 0.91 | 0.43 |
| Sham –  Anodal | 0.66 | 2.02 | 0.32 | 0.944 | 0.33 | 2.68 | 2.02 | 1.33 | 0.392 | 1.34 |

## **Left Hip Joint**

**Table 13** Post-hoc comparisons between time points for each group (Results are averaged over the level of Sound). Each comparison has an Estimate (Estimated difference) standard error (SE), t-value, p-value, and Cohen’s d. Significant contrasts are indicated by asterisks *.

|  |  | **Estimate** | **SE** | **t** | **p** | **Cohen’s d** |
| --- | --- | --- | --- | --- | --- | --- |
| Pre-Post | Control | -0.65 | 0.57 | -1.14 | 0.257 | -0.38 |
|  | Sham | -0.18 | 0.54 | -0.33 | 0.746 | -0.10 |
|  | Anodal | 1.49 | 0.53 | 2.82 | 0.0061* | 0.87 |

**Table 14** Post-hoc comparisons between groups for both time points (Results are averaged over the level of Sound). Each comparison has an Estimate (Estimated difference) standard error (SE), t-value, p-value, and Cohen’s d. Significant contrasts are indicated by asterisks *.

|  | **Pre** | | | |  | **Post** | | | |  |
| --- | --- | --- | --- | --- | --- | --- | --- | --- | --- | --- |
|  | **Estimate** | **SE** | **t** | **p** | **Cohen’s d** | **Estimate** | **SE** | **t** | **p** | **Cohen’s d** |
| Control –  Sham | -2.55 | 2.09 | -1.22 | 0.451 | -1.49 | -2.07 | 2.09 | -0.99 | 0.588 | -1.21 |
| Control –  Anodal | -0.72 | 2.05 | -0.35 | 0.934 | -0.42 | 1.43 | 2.05 | 0.70 | 0.768 | 0.83 |
| Sham –  Anodal | 1.83 | 1.99 | 0.92 | 0.633 | 1.07 | 3.50 | 1.99 | 1.76 | 0.201 | 2.04 |

## **Right Knee Joint**

**Table 15** Post-hoc comparisons between time points for all participants (Results are averaged over the levels of Sound and Group). Each comparison has an Estimate (Estimated difference) standard error (SE), t-value, p-value, and Cohen’s d. Significant contrasts are indicated by asterisks *.

|  | **Estimate** | **SE** | **t** | **p** | **Cohen’s d** |
| --- | --- | --- | --- | --- | --- |
| Pre-Post | 2.32 | 0.65 | 3.60 | 0.0006* | 0.66 |

## **Right Ankle Joint**

**Table 16** Post-hoc comparisons between time points for all participants (Results are averaged over the levels of Sound and Group). Each comparison has an Estimate (Estimated difference) standard error (SE), t-value, p-value, and Cohen’s d. Significant contrasts are indicated by asterisks *.

|  | **Estimate** | **SE** | **t** | **p** | **Cohen’s d** |
| --- | --- | --- | --- | --- | --- |
| Pre-Post | 0.16 | 0.07 | 2.32 | 0.0227* | 0.43 |

## **Left Ankle Joint**

**Table 17** Post-hoc comparisons between time points for each group (Results are averaged over the level of Sound). Each comparison has an Estimate (Estimated difference) standard error (SE), t-value, p-value, and Cohen’s d. Significant contrasts are indicated by asterisks *.

|  |  | **Estimate** | **SE** | **t** | **p** | **Cohen’s d** |
| --- | --- | --- | --- | --- | --- | --- |
| Pre-Post | Control | -0.22 | 0.07 | -3.30 | 0.0015* | -1.10 |
|  | Sham | 0.09 | 0.06 | 1.38 | 0.171 | 0.44 |
|  | Anodal | 0.07 | 0.06 | 1.18 | 0.243 | 0.36 |

**Table 18** Post-hoc comparisons between groups in both time points (Results are averaged over the level of Sound). Each comparison has an Estimate (Estimated difference) standard error (SE), t-value, p-value, and Cohen’s d. Significant contrasts are indicated by asterisks *.

|  | **Pre** | | | |  | **Post** | | | |  |
| --- | --- | --- | --- | --- | --- | --- | --- | --- | --- | --- |
|  | **Estimate** | **SE** | **t** | **p** | **Cohen’s d** | **Estimate** | **SE** | **t** | **p** | **Cohen’s d** |
| Control - Sham | -0.16 | 0.15 | -1.07 | 0.539 | -0.81 | 0.15 | 0.15 | 0.96 | 0.606 | 0.73 |
| Control - Anodal | 0.12 | 0.15 | 0.83 | 0.686 | 0.62 | 0.42 | 0.15 | 2.81 | 0.0223* | 2.08 |
| Sham-Anodal | 0.29 | 0.15 | 1.98 | 0.134 | 1.43 | 0.27 | 0.15 | 1.88 | 0.161 | 1.35 |

**BACKWARD PERTURBATION**

**Right Knee Joint**

**Table 19** Post-hoc comparisons between time points for all participants (Results are averaged over the levels of Sound and Group). Each comparison has an Estimate (Estimated difference) standard error (SE), t-value, p-value, and Cohen’s d. Significant contrasts are indicated by asterisks *.

|  | **Estimate** | **SE** | **t** | **p** | **Cohen’s d** |
| --- | --- | --- | --- | --- | --- |
| Pre-Post | -0.53 | 0.15 | -3.66 | 0.0005* | -0.68 |

**Left Knee Joint**

**Table 20** Post-hoc comparisons between time points for all participants (Results are averaged over the levels of Sound and Group). Each comparison has an Estimate (Estimated difference) standard error (SE), t-value, p-value, and Cohen’s d. Significant contrasts are indicated by asterisks *.

|  | **Estimate** | **SE** | **t** | **p** | **Cohen’s d** |
| --- | --- | --- | --- | --- | --- |
| Pre-Post | -0.48 | 0.12 | -4.14 | 0.0001* | -0.76 |

# **N1 AMPLITUDE**

**BACKWARD PERTURBATION**

**Table 21** Post-hoc comparisons between time points for all participants (Results are averaged over the levels of Sound and Group). Each comparison has an Estimate (Estimated difference) standard error (SE), t-value, p-value, and Cohen’s d. Significant contrasts are indicated by asterisks *.

|  | **Estimate** | **SE** | **t** | **p** | **Cohen’s d** |
| --- | --- | --- | --- | --- | --- |
| Pre-Post | 1.42 | 0.48 | 2.98 | 0.0038* | -0.52 |

**Table 22** Post-hoc comparisons between sound conditions for all participants (Results are averaged over the levels of Time and Group). Each comparison has an Estimate (Estimated difference) standard error (SE), t-value, p-value, and Cohen’s d. Significant contrasts are indicated by asterisks *.

|  | **Estimate** | **SE** | **t** | **p** | **Cohen’s d** |
| --- | --- | --- | --- | --- | --- |
| No Sound - Sound | 1.63 | 0.48 | 3.4 | 0.001* | -0.59 |

**Table 23** Post-hoc comparisons between groups in both time points (Results are averaged over the level of Sound). Each comparison has an Estimate (Estimated difference) standard error (SE), t-value, p-value, and Cohen’s d. Significant contrasts are indicated by asterisks *.

|  | **Estimate** | **SE** | **t** | **p** | **Cohen’s d** |
| --- | --- | --- | --- | --- | --- |
| Control - Sham | -9.21 | 3.61 | -2.55 | 0.0412* | 3.36 |
| Control - Anodal | -2.59 | 3.54 | -0.73 | 0.75 | 0.94 |
| Sham-Anodal | 6.62 | 3.45 | 1.92 | 0.15 | -2.41 |

# **N1 LATENCY**

**FORWARD PERTURBATION**

**Table 24** Post-hoc comparisons between time points for all participants (Results are averaged over the levels of Sound and Group). Each comparison has an Estimate (Estimated difference) standard error (SE), t-value, p-value, and Cohen’s d. Significant contrasts are indicated by asterisks *.

|  | **Estimate** | **SE** | **t** | **p** | **Cohen’s d** |
| --- | --- | --- | --- | --- | --- |
| Pre-Post | 4.30 | 0.70 | 6.14 | <0.0001* | 1.07 |

**BACKWARD PERTURBATION**

**Table 25** Post-hoc comparisons between time points for all participants (Results are averaged over the levels of Sound and Group). Each comparison has an Estimate (Estimated difference) standard error (SE), t-value, p-value, and Cohen’s d. Significant contrasts are indicated by asterisks *.

|  | **Estimate** | **SE** | **t** | **p** | **Cohen’s d** |
| --- | --- | --- | --- | --- | --- |
| Pre-Post | 3.93 | 0.95 | 4.13 | 0.0001* | 0.72 |

**Table 26** Post-hoc comparisons between time points for each group (Results are averaged over the level of Sound). Each comparison has an Estimate (Estimated difference) standard error (SE), t-value, p-value, and Cohen’s d. Significant contrasts are indicated by asterisks *.

|  |  | **Estimate** | **SE** | **t** | **p** | **Cohen’s d** |
| --- | --- | --- | --- | --- | --- | --- |
| Pre-Post | Control | 3.00 | 1.72 | 1.74 | 0.0848 | 0.55 |
|  | Sham | 1.45 | 1.64 | 0.89 | 0.378 | 0.27 |
|  | Anodal | 7.33 | 1.57 | 4.67 | <0.0001* | 1.35 |

**Table 27** Post-hoc comparisons between groups in both time points (Results are averaged over the level of Sound). Each comparison has an Estimate (Estimated difference) standard error (SE), t-value, p-value, and Cohen’s d. Significant contrasts are indicated by asterisks *.

|  | **Pre** | | | | | **Post** | | | | |
| --- | --- | --- | --- | --- | --- | --- | --- | --- | --- | --- |
|  | Estimate | SE | t | p | Cohen’s d | Estimate | SE | t | p | Cohen’s d |
| Control - Sham | -1.65 | 4.12 | -0.40 | 0.915 | -0.30 | -3.20 | 4.12 | -0.78 | 0.72 | -0.59 |
| Control - Anodal | -2.70 | 4.04 | -0.67 | 0.783 | -0.50 | 1.63 | 4.04 | 0.40 | 0.914 | 0.30 |
| Sham-Anodal | -1.05 | 3.94 | -0.27 | 0.962 | -0.19 | 4.83 | 3.94 | 1.23 | 0.445 | 0.89 |

# **EEG FREQUENCY BAND POWER (Cz)**

## **5.1 Averaged Theta Power**

**FORWARD PERTURBATION**

**Table 28** Post-hoc comparisons between time points for all participants (Results are averaged over the levels of Sound and Group). Each comparison has an Estimate (Estimated difference) standard error (SE), t-value, p-value, and Cohen’s d. Significant contrasts are indicated by asterisks *.

|  | **Estimate** | **SE** | **t** | **p** | **Cohen’s d** |
| --- | --- | --- | --- | --- | --- |
| Pre-Post | 0.66 | 0.15 | 4.46 | <0.0001* | 0.78 |

**Table 29** Post-hoc comparisons between sound conditions for all participants (Results are averaged over the levels of Time and Group). Each comparison has an Estimate (Estimated difference) standard error (SE), t-value, p-value, and Cohen’s d. Significant contrasts are indicated by asterisks *.

|  | **Estimate** | **SE** | **t** | **p** | **Cohen’s d** |
| --- | --- | --- | --- | --- | --- |
| No Sound - Sound | 0.30 | 0.15 | 1.99 | 0.0496* | 0.3 |

**BACKWARD PERTURBATION**

**Table 30** Post-hoc comparisons between sound conditions for all participants (Results are averaged over the levels of Time and Group). Each comparison has an Estimate (Estimated difference) standard error (SE), t-value, p-value, and Cohen’s d. Significant contrasts are indicated by asterisks *.

|  | **Estimate** | **SE** | **t** | **p** | **Cohen’s d** |
| --- | --- | --- | --- | --- | --- |
| No Sound-Sound | 0.46 | 0.15 | 3.0 | 0.0035* | 0.52 |

**Table 31** Post-hoc comparisons between time points for each group (Results are averaged over the level of Sound). Each comparison has an Estimate (Estimated difference) standard error (SE), t-value, p-value, and Cohen’s d. Significant contrasts are indicated by asterisks *.

|  |  | **Estimate** | **SE** | **t** | **p** | **Cohen’s d** |
| --- | --- | --- | --- | --- | --- | --- |
| Pre-Post | Control | -0.48 | 0.28 | -1.72 | 0.0888 | -0.54 |
|  | Sham | 0.67 | 0.27 | 2.53 | 0.0132* | 0.76 |
|  | Anodal | 0.51 | 0.25 | 2.00 | 0.0485* | 0.58 |

**Table 32** Post-hoc comparisons between groups in both time points (Results are averaged over the level of Sound). Each comparison has an Estimate (Estimated difference) standard error (SE), t-value, p-value, and Cohen’s d. Significant contrasts are indicated by asterisks *.

|  | **Pre** | | | | | **Post** | | | | |
| --- | --- | --- | --- | --- | --- | --- | --- | --- | --- | --- |
|  | **Estimate** | **SE** | **t** | **p** | **Cohen’s d** | **Estimate** | **SE** | **t** | **p** | **Cohen’s d** |
| Control –  Sham | -1.42 | 0.85 | -1.67 | 0.232 | -1.62 | -0.27 | 0.85 | -0.32 | 0.945 | -0.31 |
| Control –  Anodal | -0.63 | 0.84 | -0.75 | 0.737 | -0.71 | 0.36 | 0.84 | 0.43 | 0.902 | 0.41 |
| Sham-  Anodal | 0.80 | 0.81 | 0.98 | 0.594 | 0.91 | 0.64 | 0.81 | 0.78 | 0.718 | 0.72 |

# **5.2 Averaged Alpha Power**

**FORWARD PERTURBATION**

**Table 33** Post-hoc comparisons between time points for each group (Results are averaged over the level of Sound). Each comparison has an Estimate (Estimated difference) standard error (SE), t-value, p-value, and Cohen’s d. Significant contrasts are indicated by asterisks *.

|  |  | **Estimate** | **SE** | **t** | **p** | **Cohen’s d** |
| --- | --- | --- | --- | --- | --- | --- |
| Pre-Post | Control | -0.37 | 0.38 | -0.98 | 0.33 | -0.31 |
|  | Sham | 0.44 | 0.36 | 1.22 | 0.227 | 0.37 |
|  | Anodal | 0.95 | 0.35 | 2.75 | 0.007* | 0.80 |

**Table 34** Post-hoc comparisons between groups in both time points (Results are averaged over the level of Sound). Each comparison has an Estimate (Estimated difference) standard error (SE), t-value, p-value, and Cohen’s d. Significant contrasts are indicated by asterisks *.

|  | | | | **Pre** | | | | | **Post** | | | |
| --- | --- | --- | --- | --- | --- | --- | --- | --- | --- | --- | --- | --- |
|  | **Estimate** | **SE** | **t** | | **p** | **Cohen’s d** | **Estimate** | **SE** | | **t** | **p** | **Cohen’s d** |
| Control –  Sham | -1.36 | 1.34 | -1.01 | | 0.575 | -1.13 | -0.54 | 1.34 | | -0.41 | 0.914 | -0.45 |
| Control –  Anodal | -1.04 | 1.31 | -0.79 | | 0.71 | -0.87 | 0.28 | 1.31 | | 0.22 | 0.974 | 0.24 |
| Sham-  Anodal | 0.31 | 1.28 | 0.25 | | 0.967 | 0.26 | 0.83 | 1.28 | | 0.65 | 0.795 | 0.69 |

**BACKWARD PERTURBATION**

**Table 35** Post-hoc comparisons between sound conditions for all participants (Results are averaged over the levels of Time and Group). Each comparison has an Estimate (Estimated difference) standard error (SE), t-value, p-value, and Cohen’s d. Significant contrasts are indicated by asterisks *.

|  | **Estimate** | **SE** | **t** | **p** | **Cohen’s d** |
| --- | --- | --- | --- | --- | --- |
| No Sound - Sound | 0.59 | 0.19 | 3.03 | 0.0032* | 0.53 |

# **INTRACORTICAL EXCITABILITY**

## **6.1 Short-Interval Intracortical Inhibition (SICI)**

**Table 36** Post-hoc comparisons between muscles for all participants (Results are averaged over the levels of Time and Group). Each comparison has an Estimate (Estimated difference) standard error (SE), t-value, p-value, and Cohen’s d. Significant contrasts are indicated by asterisks *.

| **Muscle Comparisons** | **Estimate** | **SE** | **t** | **p** | **Cohen’s d** |
| --- | --- | --- | --- | --- | --- |
| Right Tibialis Anterior – Right Soleus | -0.02 | 0.03 | -0.73 | 0.884 | -0.13 |
| Right Tibialis Anterior – Left Tibialis Anterior | 0.08 | 0.03 | 2.56 | 0.0545 | 0.44 |
| Right Tibialis Anterior – Left Soleus | 0.00 | 0.03 | -0.06 | 0.999 | -0.01 |
| Right Soleus – Left Tibialis Anterior | 0.10 | 0.03 | 3.29 | 0.0064* | 0.56 |
| Right Soleus – Left Soleus | 0.02 | 0.03 | 0.67 | 0.909 | 0.11 |
| Left Tibialis Anterior – Left Soleus | -0.08 | 0.03 | -2.62 | 0.0462* | -0.45 |

## **6.2 Intracortical Facilitation (ICF)**

**Table 37** Post-hoc comparisons between muscles for all participants (Results are averaged over the levels of Time and Group). Each comparison has an Estimate (Estimated difference) standard error (SE), t-value, p-value, and Cohen’s d. Significant contrasts are indicated by asterisks *.

| **Muscle Comparisons** | **Estimate** | **SE** | **t** | **p** | **Cohen’s d** |
| --- | --- | --- | --- | --- | --- |
| Right Tibialis Anterior – Right Soleus | 0.27 | 0.09 | 3.09 | 0.0119* | -0.14 |
| Right Tibialis Anterior – Left Tibialis Anterior | 0.05 | 0.09 | 0.64 | 0.920 | 0.36 |
| Right Tibialis Anterior – Left Soleus | 0.22 | 0.09 | 2.55 | 0.0554 | -0.09 |
| Right Soleus – Left Tibialis Anterior | -0.21 | 0.09 | -2.46 | 0.0696 | 0.50 |
| Right Soleus – Left Soleus | -0.05 | 0.09 | -0.54 | 0.948 | 0.05 |
| Left Tibialis Anterior – Left Soleus | 0.17 | 0.09 | 1.91 | 0.225 | -0.45 |

**Table 38** Post-hoc comparisons between muscles for all participants between both time points (Results are averaged over the level of Group). Each comparison has an Estimate (Estimated difference) standard error (SE), t-value, p-value, and Cohen’s d. (TA: Tibialis Anterior, SOL: Soleus). Significant contrasts are indicated by asterisks *.

|  | **Pre** | | | | | **Post** | | | | |
| --- | --- | --- | --- | --- | --- | --- | --- | --- | --- | --- |
| **Muscle Comparisons** | **Estimate** | **SE** | **t** | **p** | **Cohen’s d** | **Estimate** | **SE** | **T** | **p** | **Cohen’s d** |
| Right TA –  Right SOL | 0.09 | 0.12 | 0.73 | 0.885 | 0.18 | 0.45 | 0.12 | 3.65 | 0.0019* | 0.88 |
| Right TA–  Left TA | -0.17 | 0.12 | -1.40 | 0.499 | -0.34 | 0.28 | 0.12 | 2.30 | 0.101 | 0.56 |
| Right TA–  Left SOL | 0.13 | 0.12 | 1.06 | 0.716 | 0.26 | 0.31 | 0.12 | 2.55 | 0.0554 | 0.62 |
| Right SOL–  Left TA | -0.26 | 0.12 | -2.13 | 0.146 | -0.52 | -0.16 | 0.12 | -1.34 | 0.537 | -0.33 |
| Right SOL –  Left SOL | 0.04 | 0.12 | 0.33 | 0.988 | 0.08 | -0.13 | 0.12 | -1.10 | 0.692 | -0.27 |
| Left TA –  Left SOL | 0.30 | 0.12 | 2.46 | 0.0693 | 0.60 | 0.03 | 0.12 | 0.25 | 0.995 | 0.06 |

**Table 39** Post-hoc comparisons between time points for each muscle (Results are averaged over the level of Group). Each comparison has an Estimate (Estimated difference) standard error (SE), t-value, p-value, and Cohen’s d. Significant contrasts are indicated by asterisks *.

|  |  | **Estimate** | **SE** | **t** | **p** | **Cohen’s d** |
| --- | --- | --- | --- | --- | --- | --- |
| Pre-Post | Right Tibialis Anterior | -0.29 | 0.12 | -2.35 | 0.0196* | -0.57 |
|  | Right Soleus | 0.07 | 0.12 | 0.56 | 0.574 | 0.14 |
|  | Left Tibialis Anterior | 0.17 | 0.12 | 1.35 | 0.177 | 0.33 |
|  | Left Soleus | -0.10 | 0.12 | -0.86 | 0.391 | -0.21 |
